# FLT3 expression and immune-associated transcriptional signatures in prostate cancer

**DOI:** 10.3389/fonc.2026.1970520

**Published:** 2026-10-23

**Authors:** Yongqiang Cui, Tianmou Song, Xingyue Wang, Zijin Wan, Lijun Mao

**Affiliations:** Department of Urology, The Affiliated Hospital of Xuzhou Medical University, Xuzhou, China

**Keywords:** cell migration, FLT3, immune infiltration, prostate cancer, single-cell RNA sequencing, tumor microenvironment

## Abstract

**Background:**

Bulk transcriptomic profiles combine signals from malignant, stromal, and immune cells. This study examined immune-associated gene expression in prostate cancer using bulk and single-cell transcriptomic data, and separately assessed the effects of FLT3 knockdown in prostate cancer cell lines.

**Methods:**

We screened 502 sample records from 497 patients and identified genes shared by ImmuneScore-associated differential expression and a weighted gene co-expression network module. Candidate genes were compared with four functional gene sets. We analyzed associations between FLT3 expression and immune-related signatures, examined single-cell expression patterns, and assessed FLT3 knockdown using two independent siRNAs in DU145 and 22Rv1 cells.

**Results:**

The screening identified 64 shared genes, and comparison with the functional gene sets prioritized FLT3 and AIM2. FLT3 expression was associated with several immune-cell signatures; associations were stronger for total T-cell and B-cell signatures than for the CD8-positive T-cell signature. Single-cell analyses suggested FLT3 expression in cells annotated as monocytes. In DU145 and 22Rv1 cells, both FLT3-targeting siRNAs reduced growth and colony formation, reduced Transwell migration and invasion readouts, and altered epithelial–mesenchymal transition markers.

**Conclusion:**

The tissue analyses support an association between FLT3 expression and immune-related transcriptional signals. The cell-line findings describe a separate *in vitro* phenotype and do not establish how FLT3 relates to lymphocyte infiltration. These findings do not establish a causal immune-regulatory mechanism or prognostic utility.

## Introduction

Prostate cancer is one of the most common malignancies in men. Its incidence has increased in China and other Asian countries (1, 2). Androgen deprivation therapy and androgen receptor-targeted agents remain central treatments for advanced disease. However, resistance often limits durable disease control (3, 4). This limitation creates a need for additional biological markers and therapeutic strategies.

The tumor microenvironment (TME) contains malignant, stromal, and immune cells, together with extracellular matrix components (5, 6). Interactions among these components influence progression, immune escape, metastasis, and treatment response (7, 8). Prostate cancer often has an immunosuppressive and relatively immune-cold TME. This feature may partly explain the limited activity of immune checkpoint blockade (9–13). Single-cell RNA sequencing (scRNA-seq) can help resolve this problem. It identifies the cellular sources of signals that remain mixed in bulk-tissue data (14–17).

FMS-like tyrosine kinase 3 (FLT3) is a type III receptor tyrosine kinase. It is expressed mainly in hematopoietic stem and progenitor cells (18–20). FLT3 ligand activates pathways that support the survival, differentiation, and proliferation of hematopoietic cells (21). FLT3 has been studied extensively in hematological malignancies. In contrast, its cellular distribution and functional relevance in prostate cancer remain uncertain.

The primary objective was to characterize immune-associated FLT3 expression in prostate cancer tissues using bulk and single-cell transcriptomic data. A secondary objective was to describe the effects of FLT3 knockdown on growth and Transwell assay readouts in two prostate cancer cell lines. These analyses address different cellular contexts. The cell-line experiments were not designed to test immune recruitment or to validate the tissue-level immune associations.

## Materials and methods

### Study design and TCGA-PRAD data

**Figure 1** summarizes the study workflow. We obtained gene-expression and clinical data for prostate adenocarcinoma from The Cancer Genome Atlas in January 2025. We calculated ImmuneScore, StromalScore, and ESTIMATEScore with ESTIMATE (22). **Supplementary Table S1** contains 502 sample records from 497 unique patients. It provides ESTIMATE-derived scores, OS time, and OS status; it does not provide PFI time or status. We used the survminer R package to define high- and low-score groups. We compared overall survival with Kaplan-Meier curves and two-sided log-rank tests. These cutoff-based analyses were exploratory. We performed all analyses in R version 4.2.1. Unless stated otherwise, we treated P < 0.05 as nominally significant.

**Figure 1:**
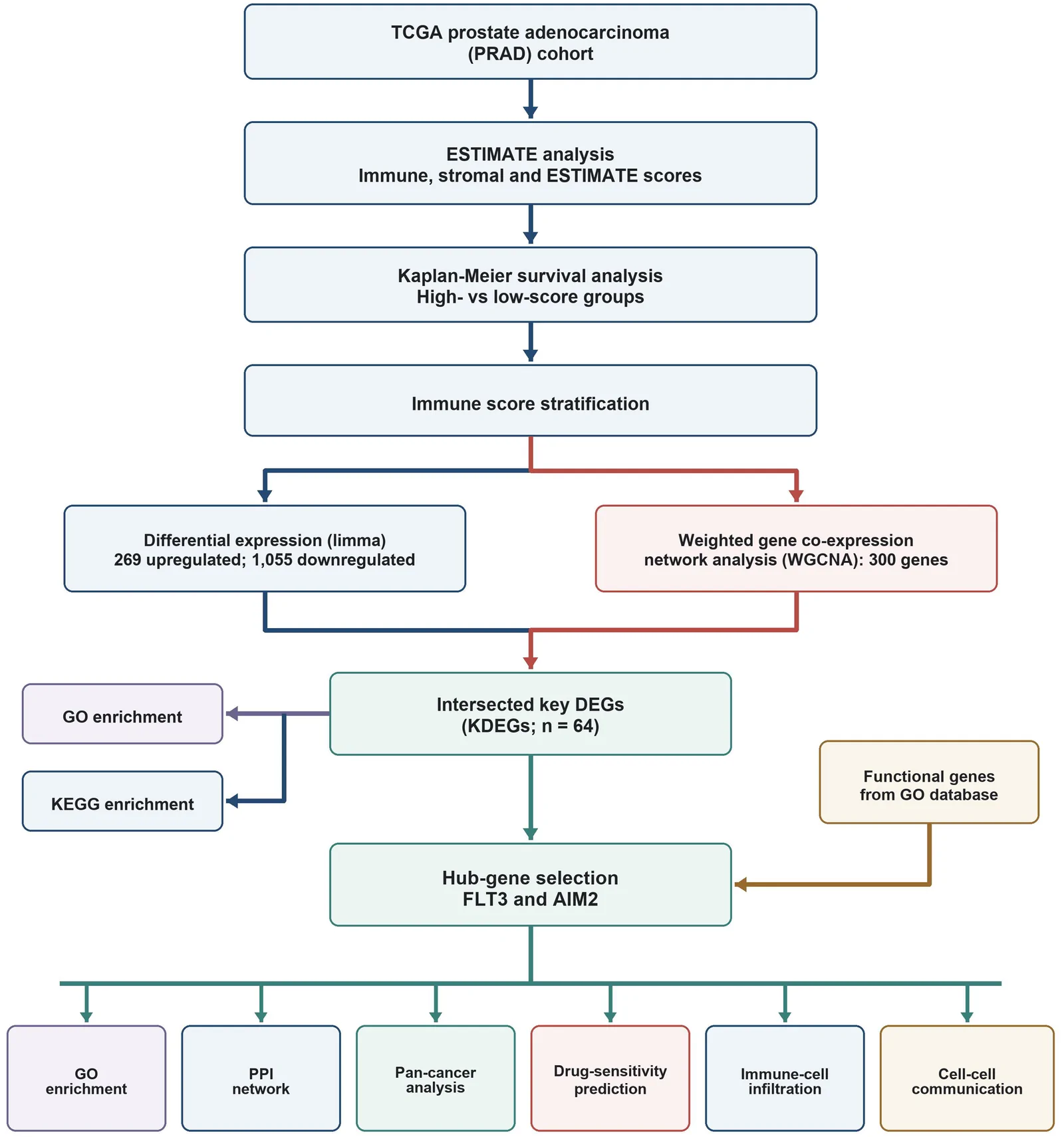
Study workflow. TCGA-PRAD transcriptomic and clinical data were analyzed with ESTIMATE, differential-expression analysis, and weighted gene co-expression network analysis (WGCNA). Candidate genes were evaluated by survival, functional enrichment, immune infiltration, pan-cancer, and predicted drug response analyses. GSE221603 single-cell RNA sequencing data were used to localize FLT3 expression and infer intercellular communication. FLT3 was subsequently silenced in DU145 and 22Rv1 cells for *in vitro* functional assessment.

### Survival outcomes and cutoff selection

We obtained overall survival (OS) and progression-free interval (PFI) data for TCGA-PRAD from UCSC Xena. We used OS for analyses of ESTIMATE-derived scores. We used PFI for gene-level analyses. We selected cutoffs with the surv_cutpoint function in survminer. This function uses maximally selected rank statistics (23). We evaluated associations with Kaplan-Meier curves and log-rank tests. We did not perform multivariable adjustment or assess independent prognostic value. Sensitivity to a median cutoff or continuous-expression modelling was not assessed. The reported cutoff-based P values are nominal. Selecting and evaluating a cutoff in the same cohort can overstate the strength of an association. These analyses are hypothesis-generating and cannot establish prognostic utility.

### Weighted gene co-expression network analysis

We ranked genes by median absolute deviation and removed the least variable 50%. We then performed weighted gene co-expression network analysis (WGCNA) (24). Pearson correlations were converted into an adjacency matrix using A_mn = |C_mn|^β. We selected a soft-thresholding power of β = 14. We used a topological overlap matrix for average-linkage hierarchical clustering. Dynamic tree cutting identified modules with a minimum size of 30 and a sensitivity of 3. We merged modules below a height of 0.25. We assigned unclassified genes to the gray module. Next, we correlated module eigengenes with ImmuneScore groups. We retained genes with an absolute module-membership value above 0.8 in the selected module.

### Candidate-gene screening

We used limma to compare gene expression between the high- and low-ImmuneScore groups (25). We retained genes with |log2 fold change| > 0.5 and nominal P < 0.05 for exploratory screening. We defined key differentially expressed genes (KDEGs) as genes shared with the ImmuneScore-associated WGCNA module. We obtained four functional gene sets from GeneCards. These sets represented glycolysis, immune escape, phosphorylation, and ferroptosis. Their intersection with the KDEGs identified FLT3 and AIM2. We then explored associations with OS, PFI, and disease-specific survival (DSS) using Kaplan-Meier analyses. The screening threshold was based on nominal P values rather than a false discovery rate (FDR) threshold. The resulting gene counts and candidate selection should therefore be interpreted as exploratory.

### Functional enrichment and protein-protein interaction analysis

We performed Gene Ontology (GO) and Kyoto Encyclopedia of Genes and Genomes (KEGG) enrichment analyses. We used clusterProfiler, enrichplot, and ggplot2 (26–30). **Supplementary Tables S9** and **S11** provide the enrichment statistics and adjusted P values. We also calculated Pearson correlations between FLT3 and all other genes in TCGA-PRAD. We retained 1, 348 genes with |r| > 0.5 and P < 0.05. For focused enrichment analysis, we selected the 25 strongest positive and negative correlates. We obtained a protein-interaction network for the 64 KDEGs from STRING. We visualized this network with Cytoscape version 3.10.3.

### Pan-cancer expression and predicted drug response

We obtained FLT3 and AIM2 expression data from harmonized TCGA, TARGET, and GTEx datasets through UCSC Xena. We transformed expression values as log2(x + 0.001). We excluded cancer types represented by fewer than three samples. We used Wilcoxon rank-sum tests for unpaired tumor-normal comparisons. We used paired signed-rank tests when matched samples were available. We obtained drug-sensitivity data from the Genomics of Drug Sensitivity in Cancer database. We used oncoPredict and ridge regression to estimate response scores from expression profiles (31). These estimates represent computational predictions, not observed clinical responses.

### Associations between FLT3 and ESTIMATE scores

FLT3 expression and ESTIMATE-derived scores were obtained for TCGA-PRAD samples. FLT3 values were transformed as log_2_(x + 0.001). Spearman correlations between FLT3 expression and ImmuneScore, StromalScore, or ESTIMATEScore were visualized with scatterplots.

### Immune-cell infiltration analyses

We estimated associations between FLT3 expression and 24 immune-cell signatures with single-sample gene set enrichment analysis (ssGSEA). We used the marker sets reported by Bindea et al. (32, 33). We calculated Spearman correlations and divided samples at the median FLT3 expression. We compared ssGSEA scores with two-sided Mann-Whitney U tests. We also analyzed TCGA-PRAD RNA sequencing data with CIBERSORT, CIBERSORTx, ssGSEA, and ESTIMATE (22, 34). Spearman coefficients summarized associations between FLT3 and the computational immune-cell estimates. We did not interpret these estimates as direct measurements of cell abundance. FLT3 was selected through an ImmuneScore-associated screen in the same TCGA-PRAD dataset. Subsequent correlations with immune estimates characterize the discovery signal and are not independent validation. The reported analyses do not separate FLT3 expression from tumor-purity effects.

### Pan-cancer immune associations

Across TCGA cancer types, FLT3 expression was correlated with chemokines, receptors, major histocompatibility complex genes, immunoinhibitors, immunostimulators, and inhibitory or stimulatory immune-checkpoint pathway genes (35). Pearson coefficients were displayed as heatmaps. These associations do not establish direct regulation by FLT3.

### Single-cell RNA sequencing analysis

We obtained the GSE221603 scRNA-seq dataset for metastatic hormone-sensitive prostate cancer (mHSPC) from the Gene Expression Omnibus (36). We processed the data with Seurat version 4.1.3 (37). We retained cells with 100–3, 000 detected genes and less than 5% mitochondrial transcripts. We normalized the data with LogNormalize and regressed out mitochondrial-transcript percentage. We then selected 2, 000 highly variable genes. Principal-component analysis preceded K-nearest-neighbor graph construction. We used Louvain clustering at a resolution of 0.5. We visualized the data with uniform manifold approximation and projection (UMAP) and t-distributed stochastic neighbor embedding (t-SNE) and assigned cell types with SingleR. Finally, we used CellChat version 1.1.3 to infer ligand-receptor communication (38). These inferred networks do not provide direct evidence of FLT3-mediated signaling.

### Cell culture, siRNA transfection and western blot analysis

DU145, 22Rv1, and RWPE-1 cells were purchased from the National Collection of Authenticated Cell Cultures, Chinese Academy of Sciences. All three cell lines were cultured in Dulbecco’s modified Eagle’s medium (DMEM; Gibco) supplemented with 10% fetal bovine serum (FBS; Gibco) at 37 °C with 5% CO_2_.We used Lipofectamine 3000 to transfect two commercially synthesized FLT3-targeting small interfering RNAs (siRNAs) or a non-targeting control. We followed the manufacturer’s protocol. We measured endogenous FLT3 protein in all three cell lines. We confirmed knockdown in DU145 and 22Rv1 cells before the functional assays.The sense-strand sequences of the two FLT3-targeting siRNAs were 5′-GAAUUUAAGUCGUGUGUUCUU-rUrU-3′ (siFLT3-1) and 5′-CGCAACAGCUUAUGGAAUUUU-rUrU-3′ (siFLT3-2). All primary antibodies used for western blotting were purchased from Proteintech. The working dilutions were 1:600 for FLT3, 1:100, 000 for E-cadherin, 1:80, 000 for Vimentin, and 1:100, 000 for GAPDH.

### Cell proliferation and colony-formation assays

Transfected DU145 and 22Rv1 cells were seeded in 96-well plates. Cell Counting Kit-8 (CCK-8) reagent was added at the time points shown in **Figure 2**, followed by incubation at 37 °C. Absorbance was measured at 450 nm. Colony formation was assessed after transfection as shown in **Figure 2D**.

**Figure 2:**
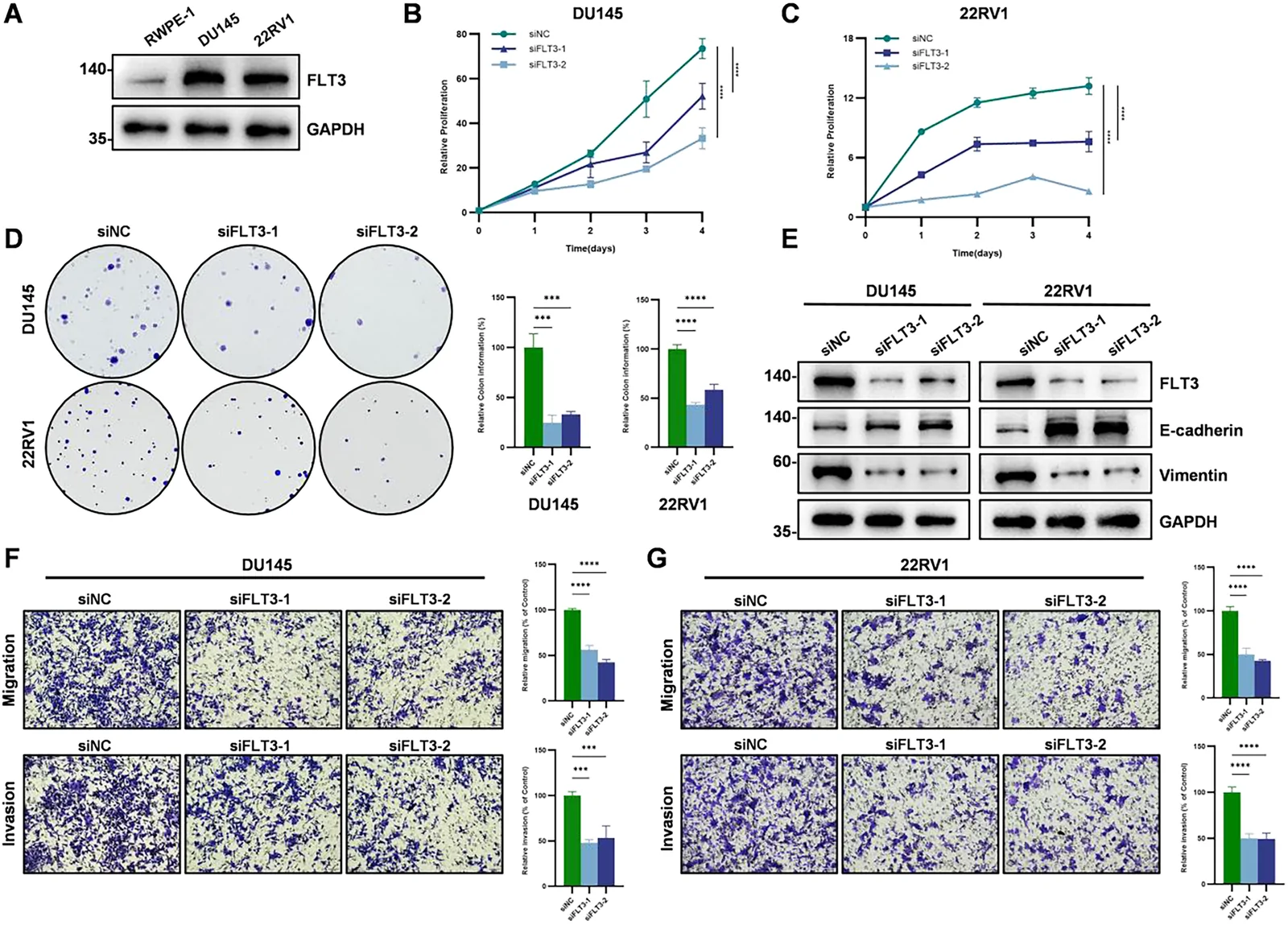
FLT3 knockdown reduces growth- and motility-associated phenotypes in prostate cancer cells. **(A)** Western blot analysis of FLT3 protein in RWPE-1, DU145, and 22Rv1 cells. **(B, C)** CCK-8 signals in DU145 **(B)** and 22Rv1 **(C)** cells after transfection with control or either of two FLT3-targeting siRNAs. **(D)** Representative colony-formation images and quantification after FLT3 knockdown. **(E)** Western blot analysis of FLT3, E-cadherin, and Vimentin after FLT3 knockdown in DU145 and 22Rv1 cells. **(F, G)** Representative Transwell images and quantification of migration and invasion in DU145 **(F)** and 22Rv1 **(G)** cells. GAPDH served as the loading control. siNC, non-targeting control siRNA; siFLT3, FLT3-targeting siRNA. For the Transwell migration and invasion assays, data are presented as mean ± SD from three independent biological replicates (n = 3 per group). Statistical comparisons were performed using ordinary one-way ANOVA followed by Dunnett’s multiple comparisons test against the siNC group. Asterisks indicate multiplicity-adjusted P values.

### Cell migration and invasion assays

For migration assays, cells were seeded in uncoated Transwell inserts. For invasion assays, cells were seeded in Matrigel-coated inserts. Cells that reached the lower membrane surface were fixed, stained, imaged, and quantified.

### Western blot analysis

Total protein was extracted, quantified, separated by sodium dodecyl sulfate–polyacrylamide gel electrophoresis (SDS-PAGE), and transferred to nitrocellulose membranes. Membranes were incubated with antibodies against FLT3, E-cadherin, Vimentin, or glyceraldehyde-3-phosphate dehydrogenase (GAPDH), followed by the corresponding secondary antibodies. Bands were visualized by enhanced chemiluminescence. Uncropped blot images are provided in **Supplementary File 1**.

### Statistical analysis

Survival associations were evaluated using Kaplan-Meier curves and two-sided log-rank tests. Wilcoxon rank-sum or paired signed-rank tests were used for tumor-normal comparisons as appropriate, and two-sided Mann-Whitney U tests were used for comparisons of ssGSEA scores. Pearson or Spearman coefficients were used for correlation analyses as specified above. Unless otherwise stated, P < 0.05 was considered nominally significant. Differential-expression and survival screening analyses were exploratory, and independent prognostic value was not assessed. The reported differential-expression screen did not use an FDR threshold. Adjusted P values for enrichment analyses do not correct the preceding candidate screen.Transwell migration and invasion experiments were performed in three independent biological replicates (n = 3 per group). Data are presented as mean ± standard deviation (SD). Group differences were assessed using ordinary one-way ANOVA followed by Dunnett’s multiple comparisons test. Each FLT3 siRNA group was compared with the negative control group. Multiplicity-adjusted P values < 0.05 were considered statistically significant.

## Results

### Exploratory associations between ESTIMATE scores and overall survival

**Supplementary Table S1** contains 502 TCGA-PRAD sample records from 497 unique patients, including 12 deaths among distinct patients. Five patients contributed two records each. Within each pair, OS time and status were identical, and all five patients were censored for OS. These records therefore did not duplicate deaths, but the table alone does not establish how repeated samples were handled in the survival models. Data-derived cutoffs defined the high- and low-score groups. The original analysis reported longer OS in the high-ImmuneScore group (nominal P = 0.011; **Figure 3A**) and a nonsignificant ESTIMATEScore comparison (P = 0.070; **Figure 3C**). The extreme StromalScore hazard ratio (HR) and unbounded confidence interval in **Figure 3B** are not interpretable as a reliable effect estimate. We do not use this estimate or its accompanying P value to infer a prognostic effect or its absence. These exploratory comparisons are limited by sparse deaths, optimized cutoffs, and unresolved patient-level model verification.

**Figure 3:**
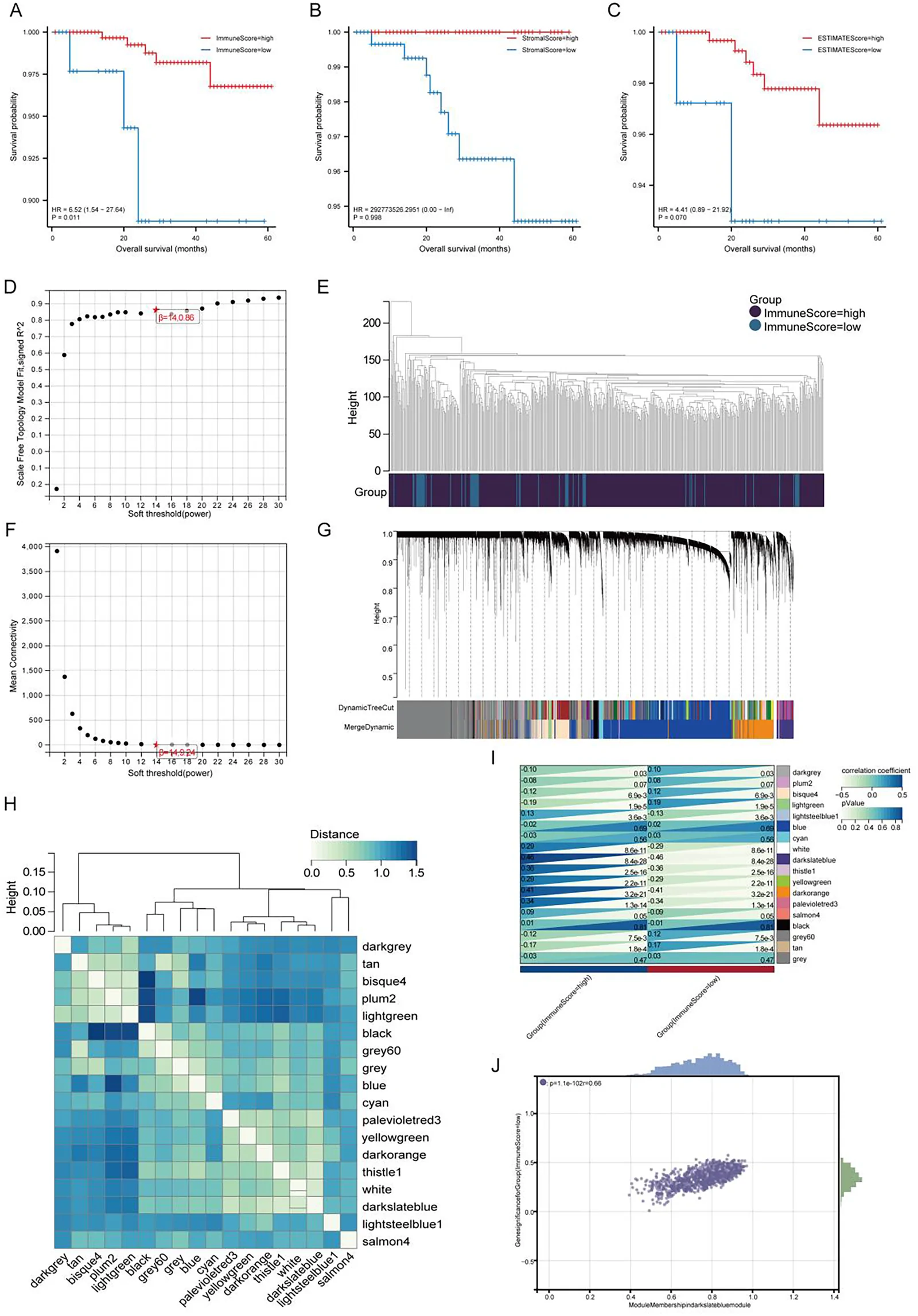
ESTIMATE-based survival analysis and WGCNA in TCGA-PRAD. **(A–C)** Kaplan-Meier overall survival curves for groups defined by ImmuneScore **(A)**, StromalScore **(B)**, and ESTIMATEScore **(C)**. The original nominal P values are displayed. The extreme HR and unbounded confidence interval in panel B are not considered reliable; the origin and validity of its accompanying P value require verification against the original model output. **(D)** Scale-free topology fit across candidate soft-thresholding powers; β = 14 was selected. **(E)** Hierarchical clustering of samples with ImmuneScore groups indicated. **(F)** Mean connectivity across candidate powers. **(G)** Gene dendrogram and module assignment before and after module merging. **(H)** Clustering heatmap of module eigengenes. **(I)** Correlations between module eigengenes and ImmuneScore groups; cells show correlation coefficients and P values. **(J)** Relationship between module membership and gene significance in the darkslateblue module. WGCNA, weighted gene co-expression network analysis; OS, overall survival.

### ImmuneScore-associated co-expression modules

A soft-thresholding power of β = 14 produced an approximately scale-free co-expression network (**Figures 3D, F**). Sample clustering displayed the ImmuneScore groups and potential outliers (**Figure 3E**). Dynamic tree cutting and module merging identified 18 modules (**Figure 3G**; **Supplementary Tables S2, S3**). Module-eigengene clustering summarized the relationships among these modules (**Figure 3H**). The darkslateblue module showed the strongest association with ImmuneScore. It contained 300 genes that met the module-membership criterion (**Figures 3I, J**; **Supplementary Table S4**).

### FLT3 and AIM2 emerge from immune-associated candidate screening

Differential-expression analysis identified 269 genes with higher expression in the high-ImmuneScore group. It also identified 1, 055 genes with lower expression (|log2 fold change| > 0.5; nominal P < 0.05; **Figure 4A**; **Supplementary Table S5**). The two gene lists mapped to immune-related and neuronal-development terms (**Supplementary Figures S1A, B**). We do not use these group-specific enrichment patterns to infer activation or suppression of immune processes. The overlap with the 300 darkslateblue-module genes yielded 64 KDEGs (**Figure 4B**; **Supplementary Table S6**). Comparison with four GeneCards-derived sets then identified FLT3 and AIM2 (**Figure 4C**; **Supplementary Tables S7, S8**).

**Figure 4:**
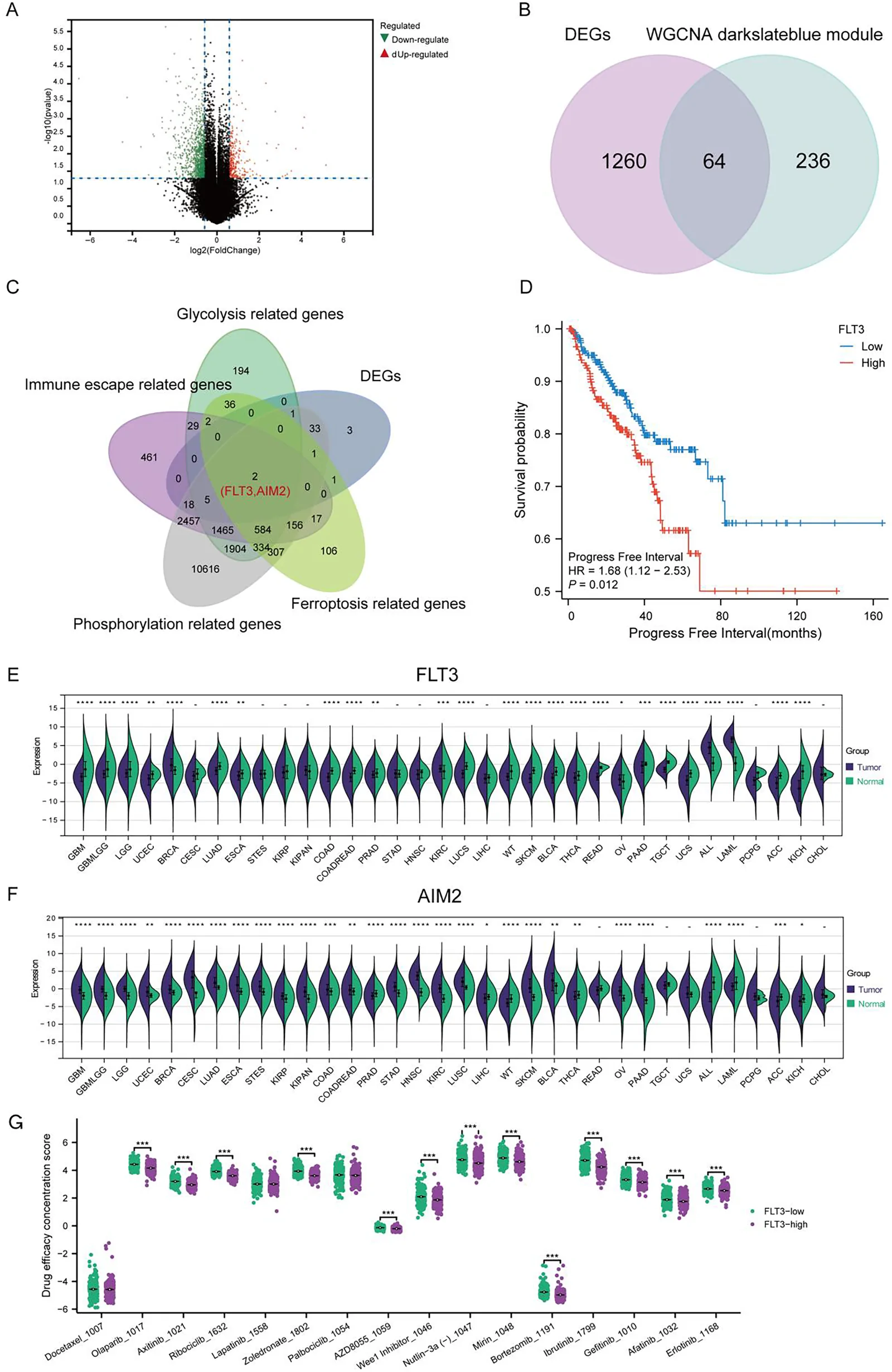
Screening and characterization of immune-associated candidate genes. **(A)** Volcano plot of genes differentially expressed between the high- and low-ImmuneScore groups using |log_2_ fold change| > 0.5 and nominal P < 0.05. **(B)** Intersection of the differentially expressed genes and the darkslateblue WGCNA module, yielding 64 key differentially expressed genes (KDEGs). **(C)** Intersection of the KDEGs with GeneCards-derived glycolysis-, immune-escape-, phosphorylation-, and ferroptosis-related gene sets, identifying FLT3 and AIM2. **(D)** Kaplan-Meier analysis of progression-free interval according to FLT3 expression. **(E, F)** FLT3 **(E)** and AIM2 **(F)** expression across tumor and normal tissues in the pan-cancer dataset. **(G)** Predicted drug response scores in the high- and low-FLT3 groups. ns, P ≥ 0.05; *P < 0.05; **P < 0.01; ***P < 0.001; ****P < 0.0001. PFI, progression-free interval.

### Exploratory survival findings for FLT3

Higher FLT3 expression was associated with shorter PFI in the unadjusted, cutoff-based analysis (nominal log-rank P = 0.012; **Figure 4D**). The supplementary analyses did not show significant FLT3 associations with OS or DSS (**Supplementary Figures S1C, D**). AIM2 was not significantly associated with PFI (**Supplementary Figure S1E**). The FLT3 PFI result used an outcome-optimized cutoff without clinical adjustment or independent validation. The available supplementary table reports OS rather than PFI events, so it cannot establish the PFI event count or the group-specific numbers at risk. Robustness to alternative cutoffs and clinical adjustment was not assessed. This finding does not establish FLT3 as an independent prognostic biomarker.

### FLT3 and AIM2 expression across cancer types

In the harmonized pan-cancer dataset, FLT3 expression was lower in prostate tumors than in normal prostate samples (**Figure 4E**). FLT3 and AIM2 also differed between tumor and normal samples in several other cancer types (**Figures 4E, F**). However, these comparisons included tissues with different cellular compositions. They therefore cannot establish tumor-cell-specific expression changes.

### Predicted drug-response differences between FLT3 groups

Predicted responses to olaparib, axitinib, ribociclib, and zoledronate differed between the FLT3 expression groups (**Figure 4G**). Predicted responses to docetaxel, lapatinib, and palbociclib did not differ. These oncoPredict results are computational estimates. They do not demonstrate clinical drug sensitivity. The analysis did not test whether FLT3 perturbation changes response to any of these drugs. It therefore provides no direct evidence that FLT3 causes the predicted response differences.

### KDEGs and FLT3-correlated genes are enriched for immune functions

GO and KEGG analyses linked the 64 KDEGs to immune processes and cytokine-receptor pathways (**Figures 5A, B, D, E**; **Supplementary Table S9**). In TCGA-PRAD, 1, 348 genes correlated with FLT3 at |Pearson r| > 0.5 and P < 0.05 (**Supplementary Table S10**). The strongest correlates were enriched for lymphocyte and T-cell differentiation terms (**Figures 5C, F**; **Supplementary Table S11**). The STRING network identified PTPRC as a highly connected gene. Its modules contained immune-associated and mitochondrial genes (**Figures 5G, H**). Together, these analyses linked FLT3 to an immune-centered transcriptional context.

**Figure 5:**
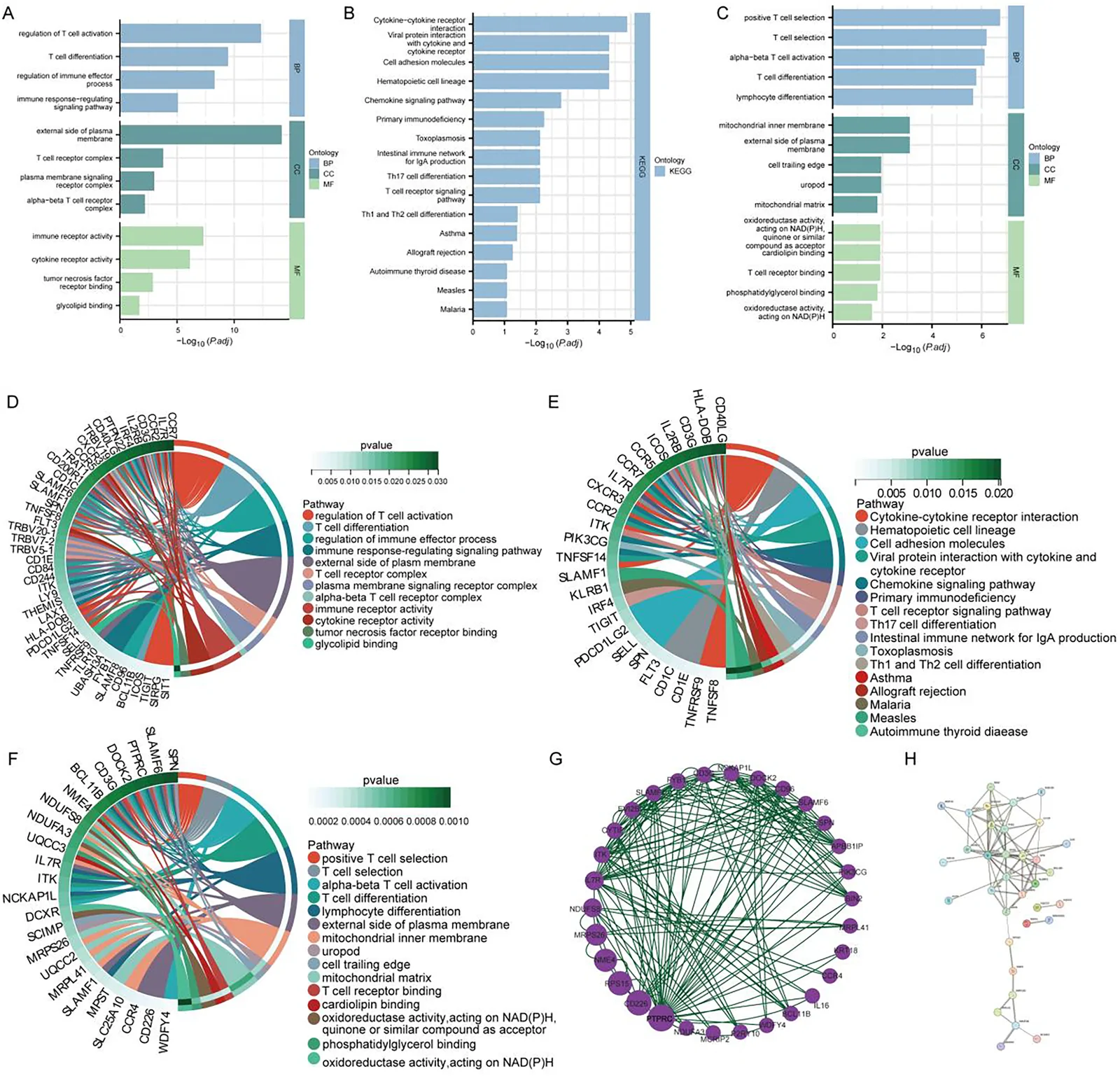
Functional enrichment and protein interaction analyses of the 64 KDEGs and FLT3-correlated genes. **(A, B)** Gene Ontology (GO) **(A)** and Kyoto Encyclopedia of Genes and Genomes (KEGG) **(B)** enrichment of the 64 KDEGs. **(C)** GO enrichment of the most strongly FLT3-correlated genes. **(D–F)** Gene-concept networks corresponding to the enrichment results in **(A–C)**, respectively. **(G)** STRING protein-protein interaction network of the KDEGs. **(H)** Network modules identified from the interaction network. BP, biological process; CC, cellular component; KDEG, key differentially expressed gene; MF, molecular function. KEGG pathway information was used with permission from Kanehisa Laboratories (https://www.kegg.jp).

### FLT3 expression covaries with immune and stromal signals

FLT3 expression correlated positively with several ssGSEA immune-cell signatures (**Supplementary Figure S2**). The correlations were stronger for total T-cell and B-cell signatures than for the CD8-positive T-cell signature. The corresponding group comparisons also showed higher T-cell and B-cell scores in the high-FLT3 group (**Supplementary Figure S3**). These findings indicate a broad lymphocyte-associated expression pattern rather than a selective association with CD8-positive T cells. FLT3 also correlated with macrophage, neutrophil, and regulatory T-cell signatures. It correlated negatively with the T helper 17 (Th17)-cell signature. Correlations with plasmacytoid dendritic cells and CD56bright natural killer cells were not significant.

FLT3 correlated with ImmuneScore (R = 0.64), StromalScore (R = 0.54), and ESTIMATEScore (R = 0.65; **Figures 6A–C**). CIBERSORT and ssGSEA identified additional associations with immune estimates (**Figures 6D–G**; **Supplementary Figures S2, S3**). The direction and magnitude varied across cancer types (**Figure 6H**). Because these TCGA-PRAD analyses reused the discovery cohort, they describe the selected immune-associated signal. They do not provide independent validation or demonstrate immune recruitment by FLT3.

**Figure 6:**
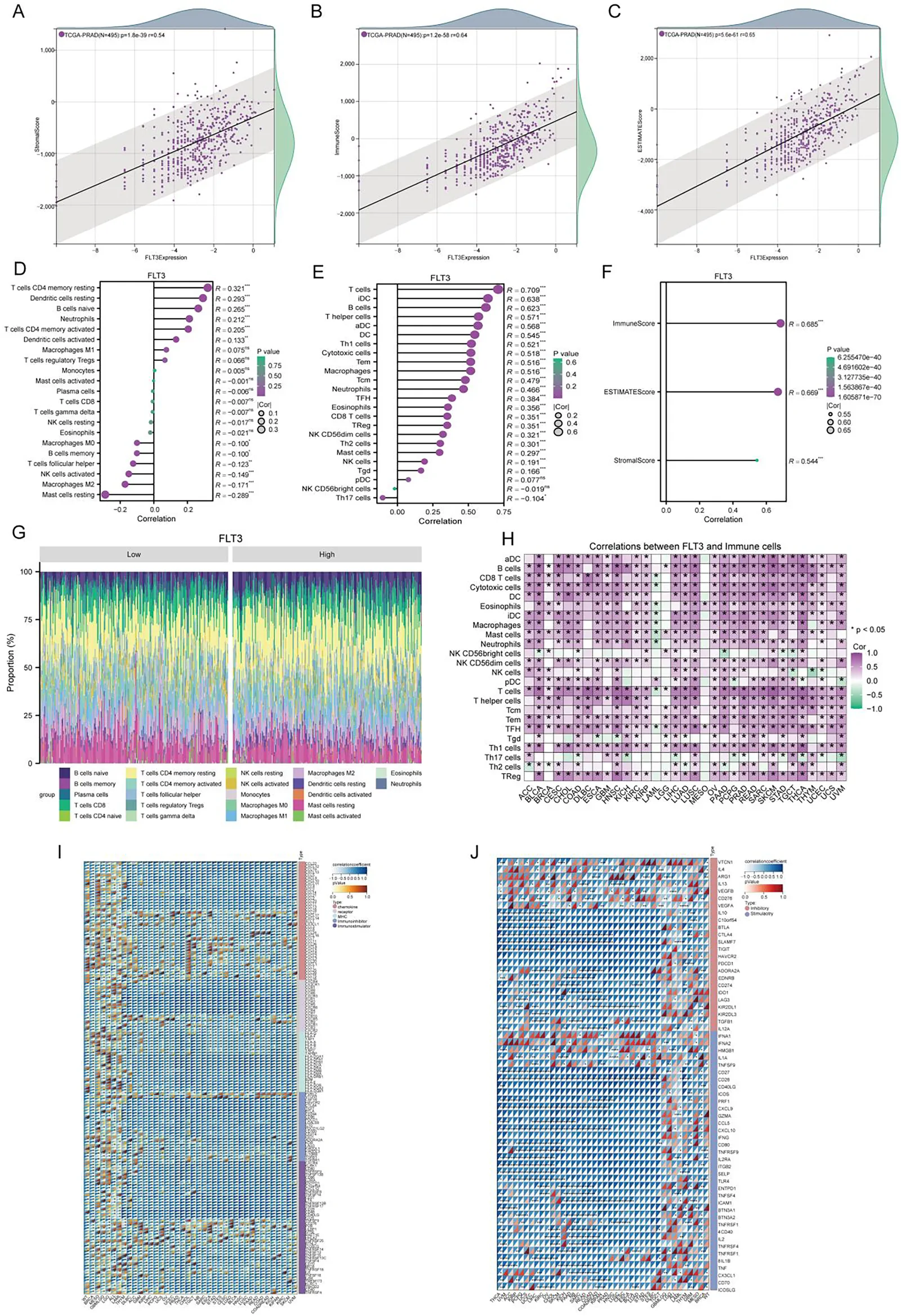
Associations of FLT3 expression with immune infiltration estimates and immune-related genes. **(A–C)** Correlations of FLT3 expression with StromalScore **(A)**, ImmuneScore **(B)**, and ESTIMATEScore **(C)** in TCGA-PRAD. **(D–F)** Associations of FLT3 expression with immune-cell estimates generated by CIBERSORT **(D)**, single-sample gene set enrichment analysis (ssGSEA) **(E)**, and ESTIMATE **(F)**. **(G)** CIBERSORTx-estimated immune-cell proportions in samples ordered by FLT3 expression. **(H)** Pan-cancer correlations between FLT3 expression and estimated immune-cell abundance. **(I)** Pan-cancer correlations between FLT3 and 150 immunomodulatory genes, comprising chemokines, chemokine receptors, major histocompatibility complex genes, immunoinhibitors, and immunostimulators. **(J)** Pan-cancer correlations between FLT3 and inhibitory or stimulatory immune-checkpoint pathway genes. Asterisks indicate the P value thresholds defined in each panel.

### FLT3 is associated with immune-regulatory genes across cancers

FLT3 correlated with many immune-regulatory and immune-checkpoint genes in PRAD and other TCGA cancers (**Figures 6I, J**). These transcriptomic associations may partly reflect immune-cell abundance and do not show that FLT3 directly regulates immune-checkpoint pathways.

### Single-cell maps suggest FLT3 expression in annotated myeloid cells

After quality control, the GSE221603 analysis retained 10, 994 cells and identified 20 transcriptional clusters. SingleR-assigned cell identities were displayed on the UMAP and t-SNE projections (**Figures 7B, E**). FLT3 expression was observed in cells annotated as monocytes (**Figures 7C, F**), suggesting a possible myeloid contribution to the tissue-level signal. These qualitative maps do not establish monocyte-specific expression, within-population FLT3-positive fractions, or consistency across individual samples. The “iPS_cells” label is a reference-derived annotation and should not be interpreted as evidence of induced pluripotent stem cells in prostate tissue.

**Figure 7:**
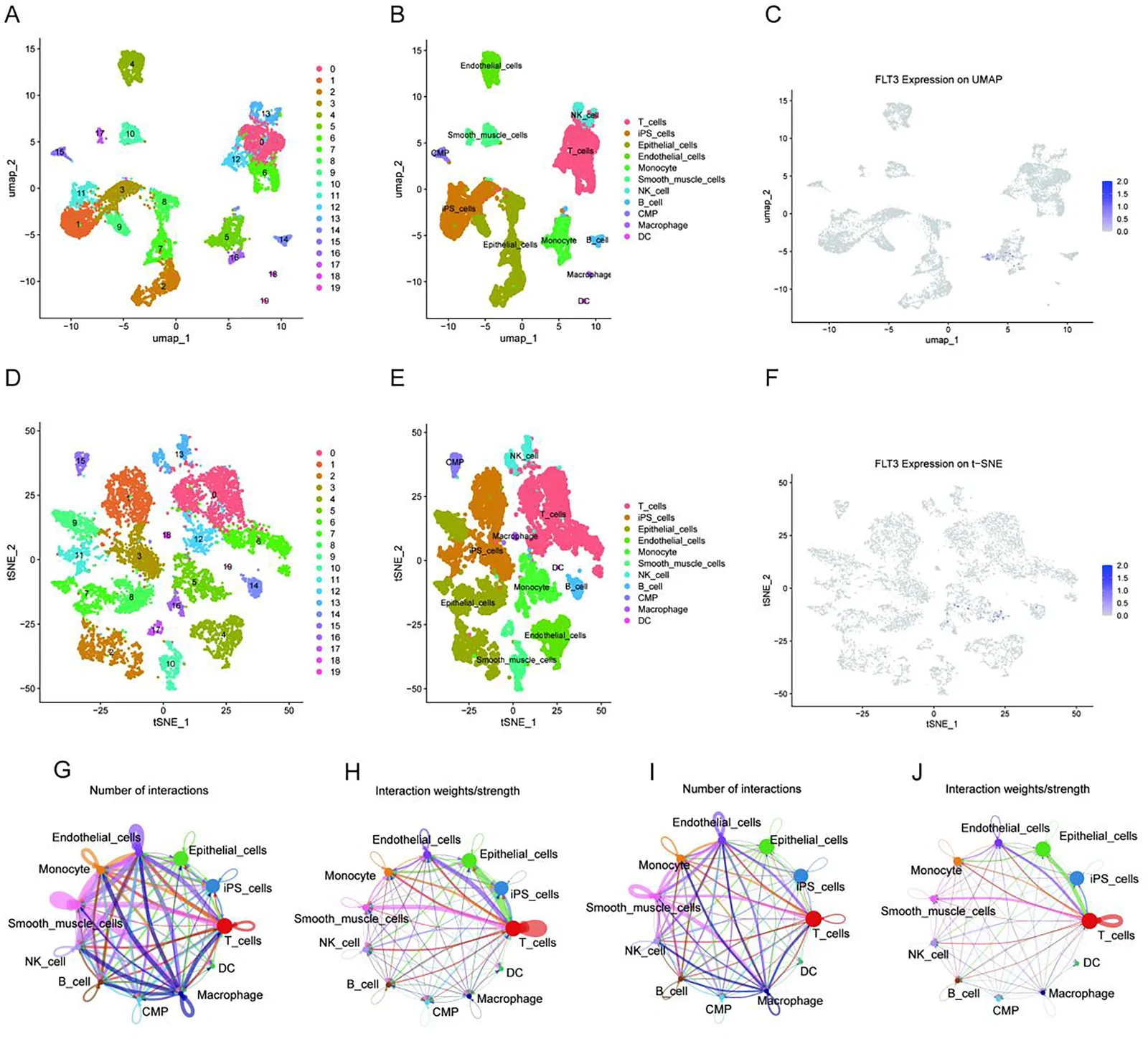
Single-cell localization of FLT3 and inferred cell-cell communication in GSE221603. **(A, D)** UMAP **(A)** and t-SNE **(D)** projections of 20 transcriptional clusters. **(B, E)** SingleR-based cell-type annotations displayed on the UMAP **(B)** and t-SNE **(E)** projections. **(C, F)** FLT3 expression across the UMAP **(C)** and t-SNE **(F)** projections. **(G, H)** Inferred number **(G)** and aggregate strength **(H)** of CellChat interactions among annotated cell populations. **(I, J)** Additional views of the inferred interaction networks. Cell identities are provisional SingleR assignments; the “iPS_cells” label is not evidence of induced pluripotent stem cells in the tissue. CMP, common myeloid progenitor; DC, dendritic cell; NK, natural killer; t-SNE, t-distributed stochastic neighbor embedding; UMAP, uniform manifold approximation and projection.

### CellChat infers communication among immune and stromal populations

The inferred number and strength of CellChat interactions differed among cell populations (**Figures 7G, H**). Under the current cell annotations, predicted networks connected monocytes with macrophages, dendritic cells, and endothelial cells. Other networks connected T cells with natural killer cells and dendritic cells (**Figures 7I, J**; **Supplementary Figures S4–S6**). FLT3 was not perturbed in this analysis. The inferred networks therefore provide cellular context but do not identify FLT3 as a communication mediator.

### FLT3 knockdown alters growth and Transwell assay readouts

Western blotting detected FLT3 protein in RWPE-1, DU145, and 22Rv1 cells. The two cancer cell lines showed stronger bands than RWPE-1 cells (**Figure 2A**). Two independent FLT3-targeting siRNAs reduced CCK-8 signals and colony formation in both cancer cell lines (**Figures 2B–D**). Knockdown also increased E-cadherin and decreased Vimentin, alongside lower Transwell migration and invasion counts (**Figures 2E–G**). These findings are consistent with altered growth and epithelial–mesenchymal transition-associated phenotypes. Because reduced growth or viability can lower the number of cells reaching the Transwell membrane, these assays do not establish a migration-specific or invasion-specific effect.

## Discussion

The principal finding is the association between FLT3 expression and immune-related transcriptional signatures in prostate cancer. The stronger correlations with total T-cell and B-cell signatures than with the CD8-positive T-cell signature suggest a broader immune-composition pattern. The cell-line experiments address a separate question: whether reducing FLT3 changes growth and Transwell assay readouts in cultured prostate cancer cells. They provide phenotypic context, rather than mechanistic validation of the tissue-level immune association.

Bulk expression reflects the mixture of cell populations within a specimen. Its association with immune signatures can therefore arise from variation in cell abundance as well as expression within individual cells (22). FLT3 was selected through an ImmuneScore-associated screen, and the subsequent TCGA-PRAD immune analyses reused that cohort. These related observations characterize the selected signal rather than provide independent validation. They cannot determine whether FLT3 promotes lymphocyte recruitment.

The single-cell maps suggest a myeloid contribution to FLT3 expression but do not resolve its distribution among monocytes, dendritic cells and progenitors with sufficient certainty to support a lineage-specific conclusion. The CellChat networks likewise describe predicted interactions under the current annotations; they do not independently validate cell identity or demonstrate FLT3-mediated immune regulation.

The tissue and cell-line findings need not represent the same biological process. A gene can contribute to a bulk immune-associated signal while showing phenotypic effects when perturbed in a cultured epithelial model. This is a possible interpretation of the two observations, not a mechanism demonstrated here. FLT3 has established functions in hematopoietic cells, and FLT3-ligand studies provide a broader immune context (39–43). In the present cell-line experiments, concordant results with two siRNAs in two models show a consistent direction of effect across the tested reagents and models. They do not replace an siRNA-resistant rescue or identify a downstream signaling pathway. Rescue and phosphorylation measurements would be needed to test target specificity and pathway dependence. Reduced growth or viability may also contribute to the Transwell findings.

Kuncman et al. similarly associated higher FLT3 expression with greater inferred immune infiltration in non-small cell lung cancer, particularly natural killer-cell and dendritic-cell signatures (44). They reported a favorable disease-free survival association in lung adenocarcinoma and a similar, nonsignificant trend in squamous carcinoma. The direction differs from the exploratory adverse PFI association in our study. Differences in cellular composition, tumor biology, and endpoint definitions may contribute. Neither study establishes a causal relationship between FLT3 expression, immune recruitment, and survival.

The disease settings also differ across our analyses. TCGA-PRAD predominantly represents primary tumors, whereas the single-cell dataset concerns mHSPC. DU145 originated from a brain metastasis (45). The 22Rv1 line arose from a xenograft that relapsed after castration and retains androgen receptor expression (46). These cell lines do not reproduce the clinical cohorts or their immune microenvironments. We therefore interpret their responses as observations in those experimental models, without assuming that they explain the bulk-tissue associations.

The outcome analyses are exploratory. FLT3 was not significantly associated with OS or DSS, and the PFI association used an outcome-optimized cutoff without clinical adjustment. The findings do not establish prognostic utility.

The drug-response predictions likewise describe associations within expression-based models. Shared transcriptional programs and differences in tissue composition could associate FLT3 with predicted response without a direct pharmacological relationship. These explanations were not tested. Because the study did not measure drug response after FLT3 perturbation, the predictions do not identify a FLT3-dependent drug mechanism or support treatment selection.

### Strengths and limitations

The combination of tissue transcriptomics and two-siRNA experiments in two cell lines provides observations across several analytical and experimental settings. The study remains exploratory. Candidate screening used nominal P values; the immune analyses reused the discovery cohort and were not adjusted for tumor purity. The handling of repeated patient records was not fully documented. Automated single-cell labels do not establish lineage-specific expression or sample-level consistency. Survival analyses were unadjusted and used optimized cutoffs. The cell experiments lacked rescue and downstream signaling measurements, and growth effects may influence Transwell results. These limitations restrict the conclusions to immune-associated expression and *in vitro* phenotypes; they preclude a demonstrated link between FLT3, immune recruitment, and clinical outcome.

## Conclusion

FLT3 expression is associated with immune-related transcriptional signatures in prostate cancer. Single-cell maps suggest a myeloid contribution to this tissue signal. Separately, FLT3 knockdown alters growth and Transwell assay readouts in two prostate cancer cell lines. The findings identify context-dependent associations and phenotypes, without establishing that tumor-cell FLT3 regulates immune infiltration or has prognostic or therapeutic utility.

## Data Availability

TCGA-PRAD data are available through the Genomic Data Commons and UCSC Xena. Single-cell RNA sequencing data are available from the Gene Expression Omnibus under accession GSE221603. Gene-level results and enrichment outputs are provided in **Supplementary Tables S1–S11**. This study does not report original computer code. Additional information required to reanalyze the reported data is available from the corresponding author upon reasonable request.
