# Supplementary material for: FLT3 expression and immune-associated transcriptional signatures in prostate cancer
Source: Front Oncol. 2026 Oct 23;16:1970520. doi: 10.3389/fonc.2026.1970520 (PMC13739570; doi:10.3389/fonc.2026.1970520)
Supplement: Supplementary file 1 [file Supplementaryfile1.pdf]

Supplementary figures

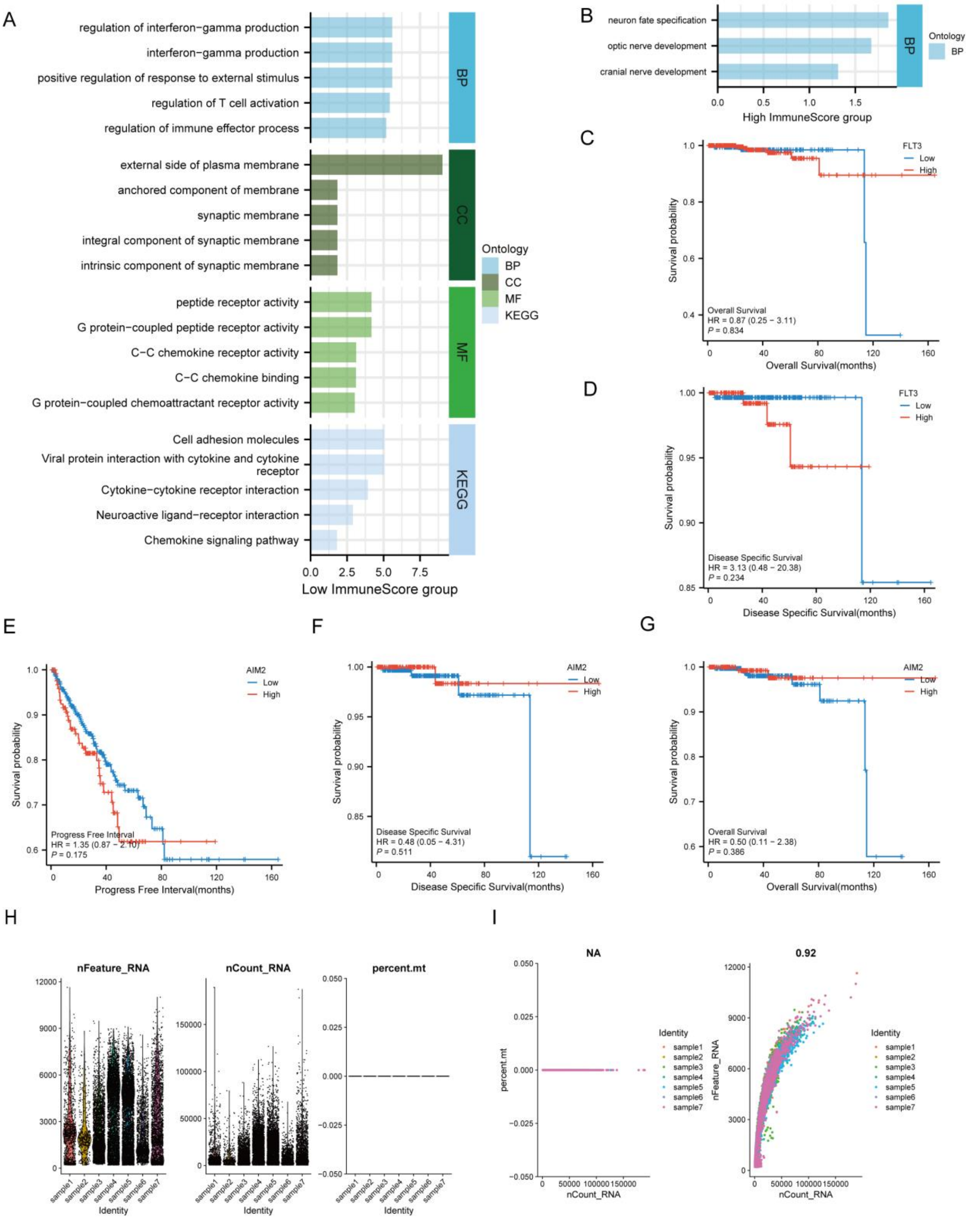

Supplementary Figure S1. Functional enrichment, survival analyses, and single-cell quality-control metrics, related to Figures 3 and 6. (A) GO and KEGG enrichment of genes expressed at higher levels in the low-ImmuneScore group. (B) GO enrichment of genes expressed at higher levels in the high-ImmuneScore group. (C–G) Kaplan-Meier analyses of overall survival (OS) and disease-specific survival (DSS) according to FLT3 expression, and progression-free interval (PFI), DSS, and OS according to AIM2 expression. (H) Distributions of nFeature_RNA, nCount_RNA, and mitochondrial-transcript percentage across the seven analyzed samples. (I) Relationships of mitochondrial-transcript percentage with nCount_RNA and of nFeature_RNA with nCount_RNA; the latter correlation coefficient is shown in the panel.

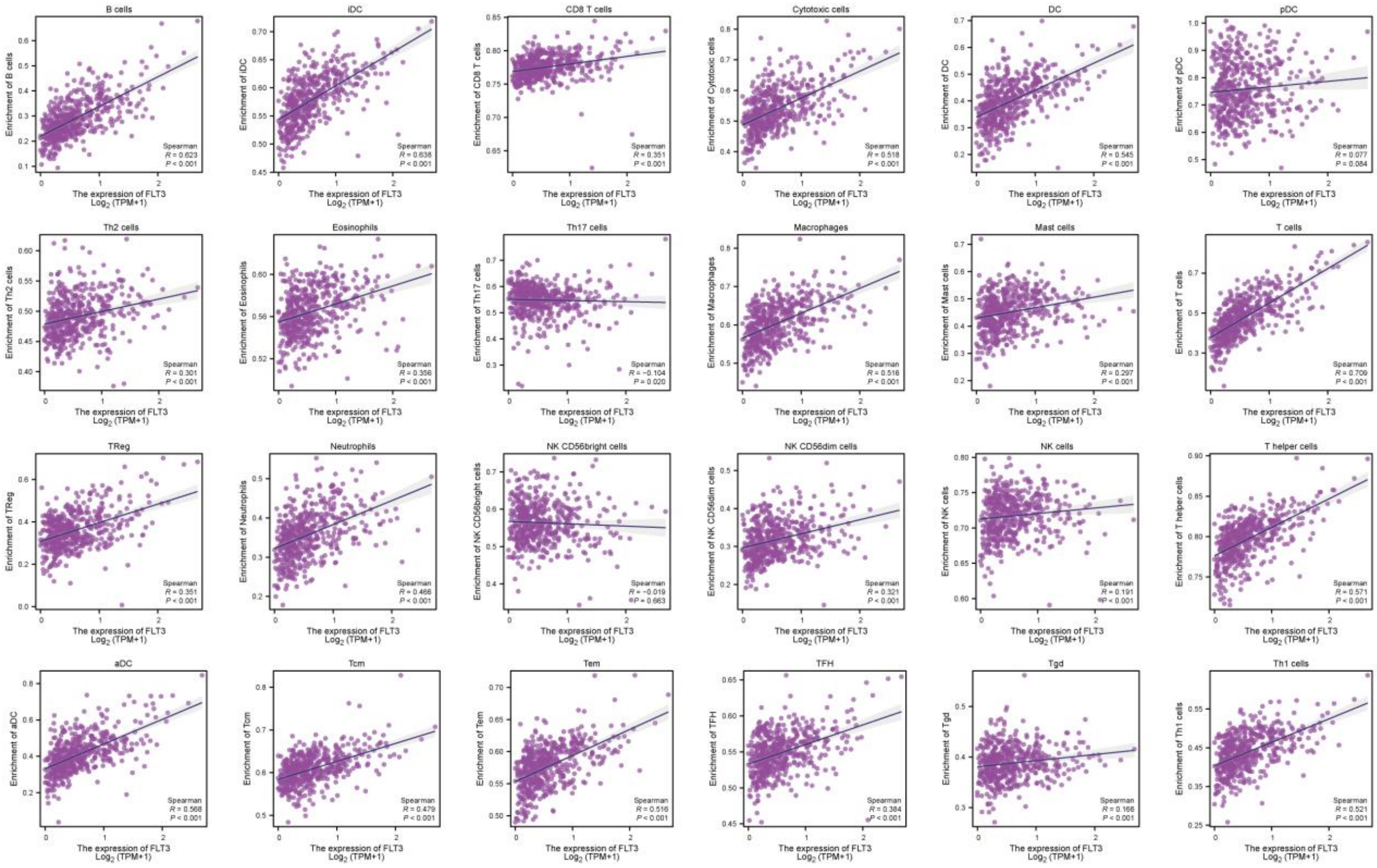

Supplementary Figure S2. Correlations between FLT3 expression and 24 ssGSEA-derived immune-cell scores in TCGA-PRAD, related to Figure 5. Each panel shows the Spearman correlation for one immune-cell signature.

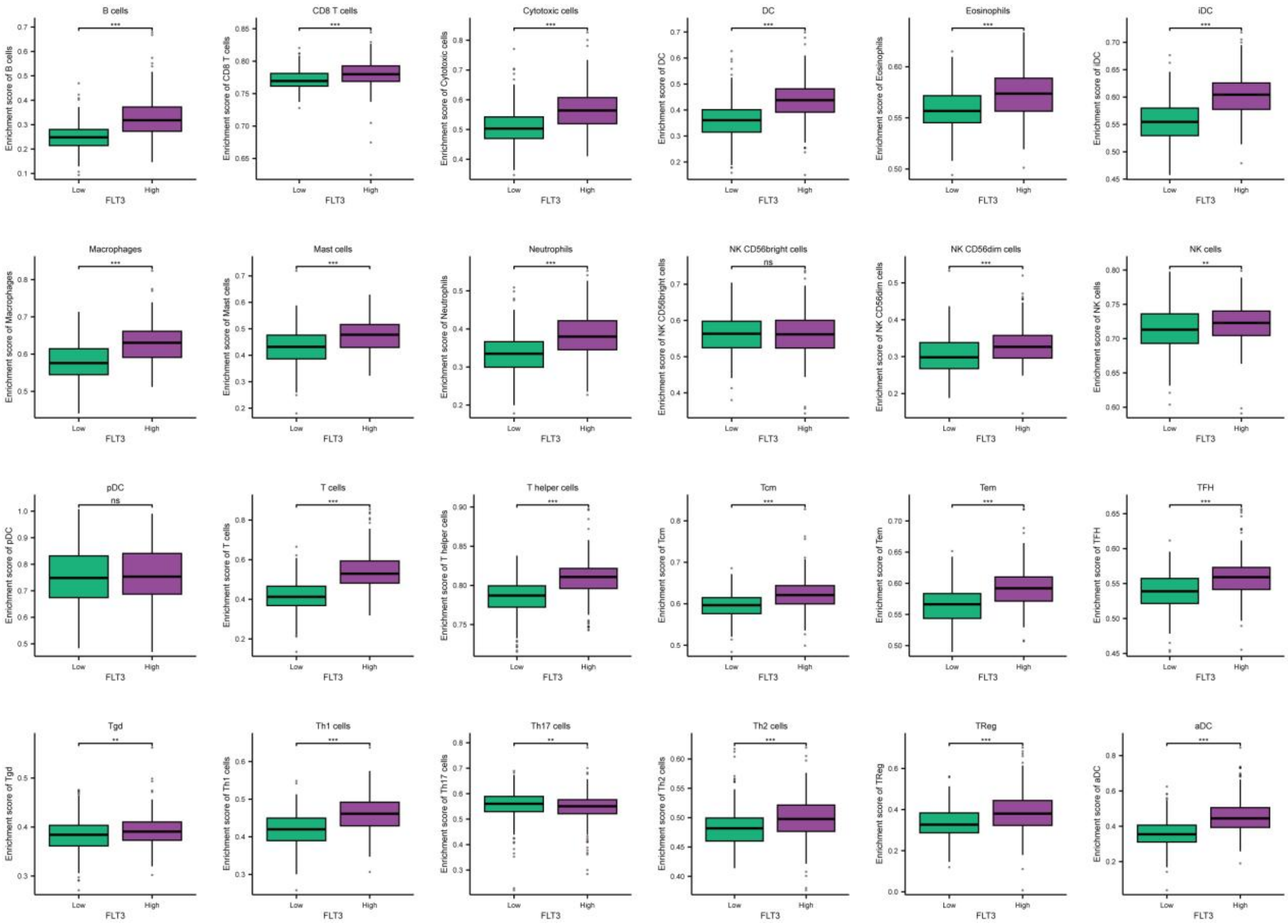

Supplementary Figure S3. Estimated immune-cell abundance in the high- and low-FLT3 expression groups, related to Figure 5. Boxplots show ssGSEA scores for 24 immune-cell signatures. ns, P ≥ 0.05; **P < 0.01; ***P < 0.001.

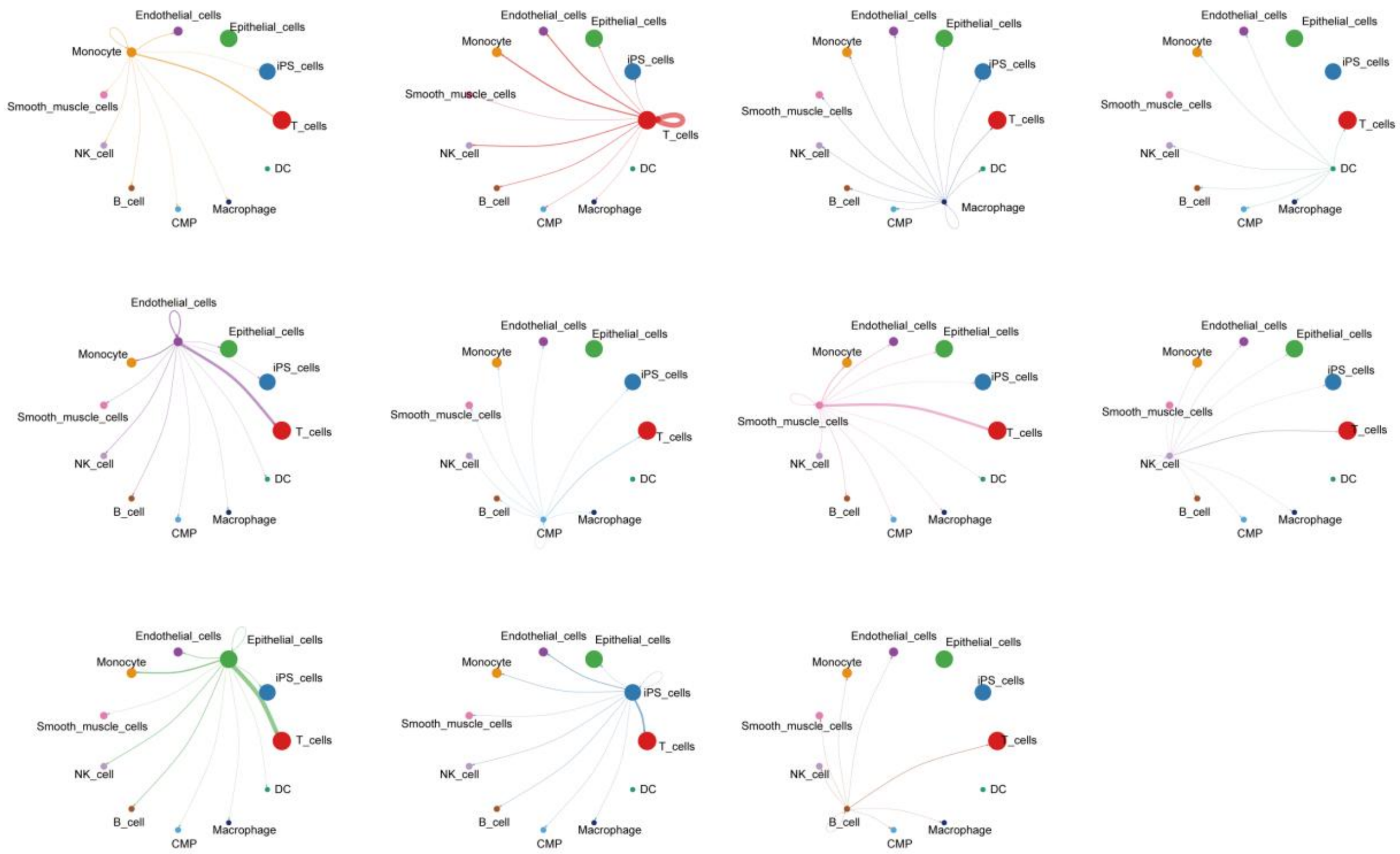

Supplementary Figure S4. CellChat-inferred signaling-pathway networks, related to Figure 6. Each panel represents one signaling pathway. Nodes denote annotated cell populations, and edge width reflects inferred interaction strength.

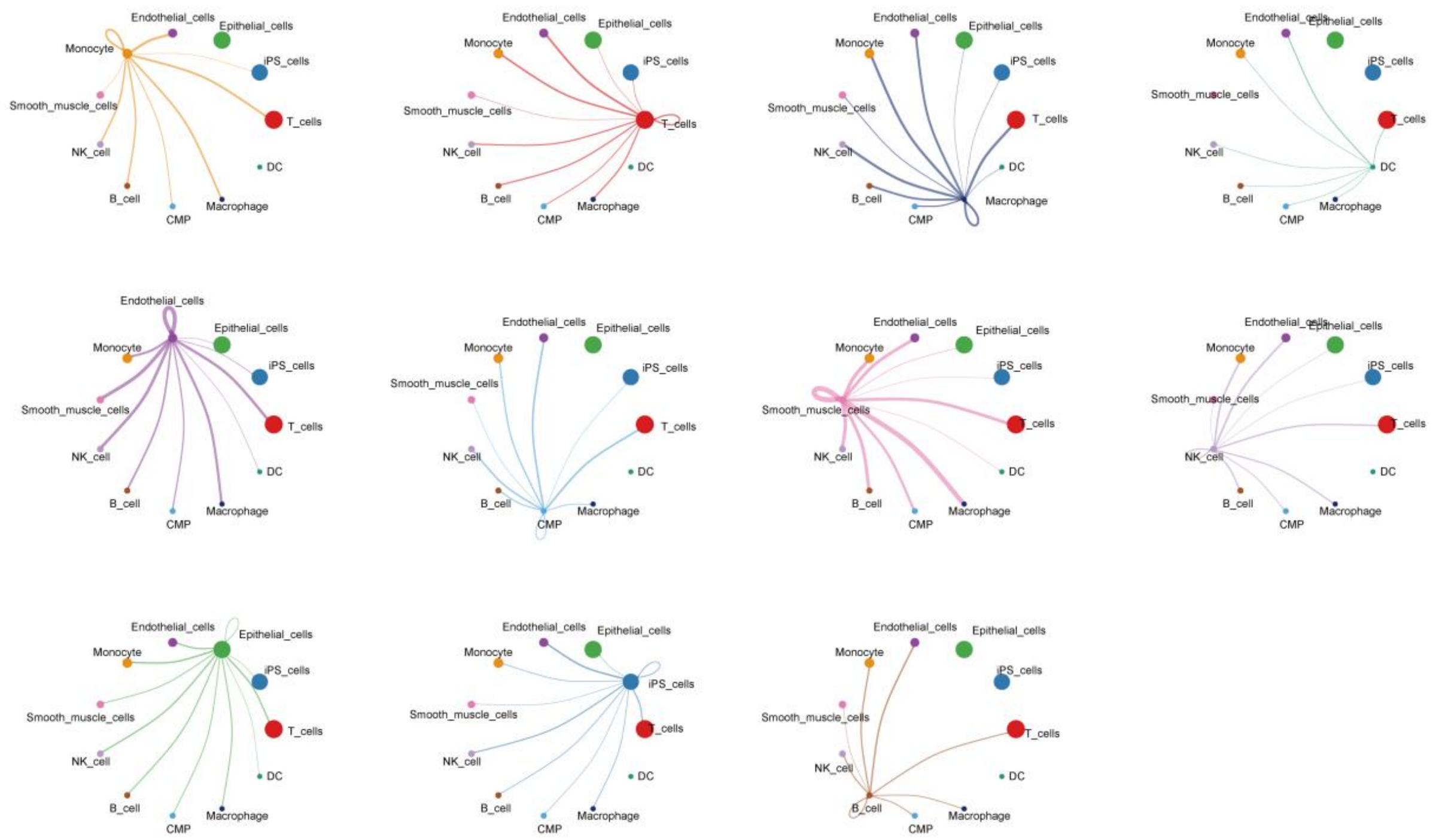

Supplementary Figure S5. CellChat-inferred outgoing communication networks by sender population, related to Figure 6. Each panel shows the predicted signals originating from one annotated cell population.

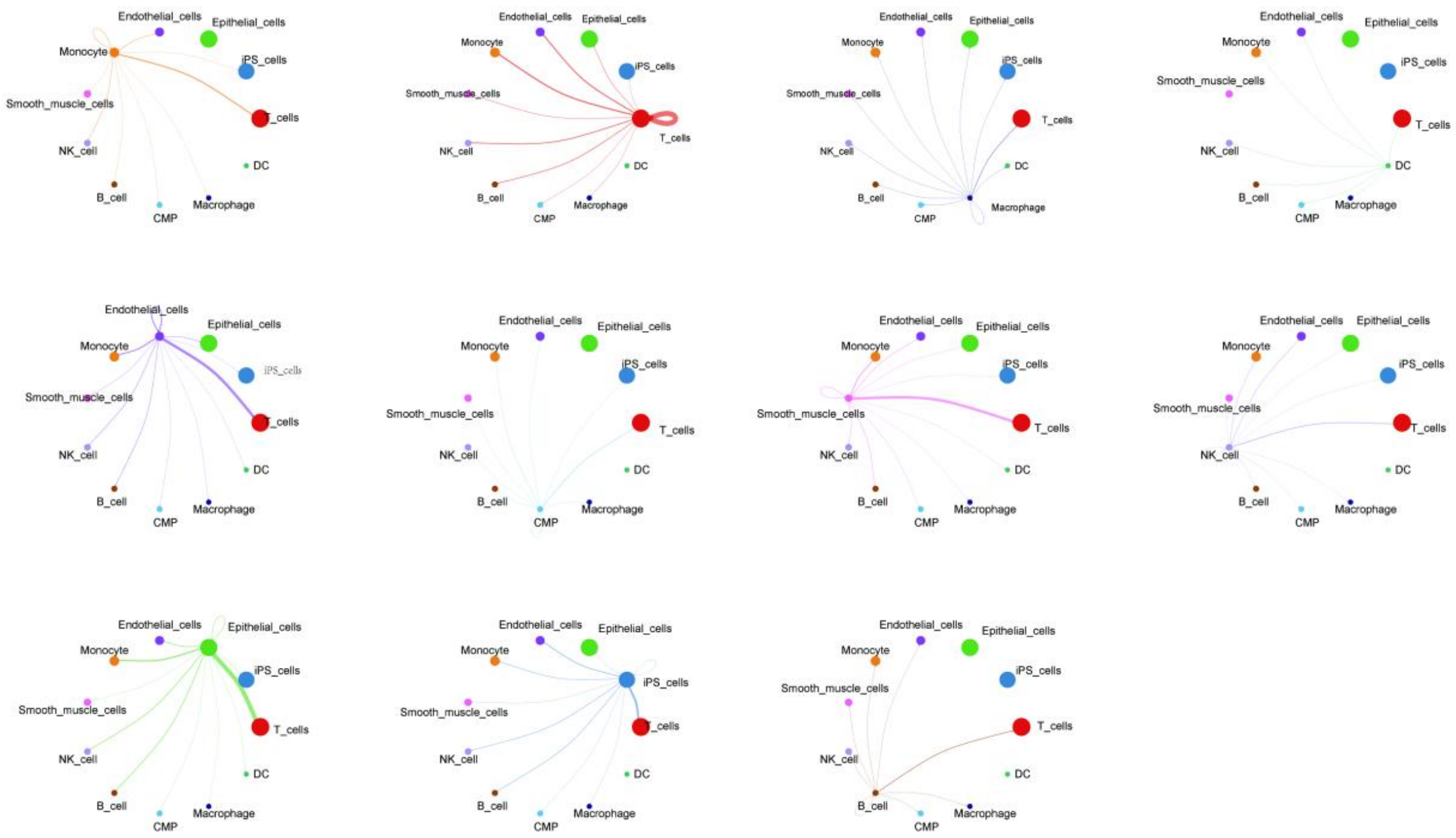

Supplementary Figure S6. CellChat-inferred incoming communication networks by receiver population, related to Figure 6. Each panel shows the predicted signals received by one annotated cell population.
