# Supplementary material for: FLT3 expression and immune-associated transcriptional signatures in prostate cancer
Source: Front Oncol. 2026 Oct 23;16:1970520. doi: 10.3389/fonc.2026.1970520 (PMC13739570; doi:10.3389/fonc.2026.1970520)
Supplement: Supplementary file 2 [file Supplementaryfile2.pdf]

Annotated western blot images corresponding to Figure 2. These images are provided for panel and lane identification.

Fig2A

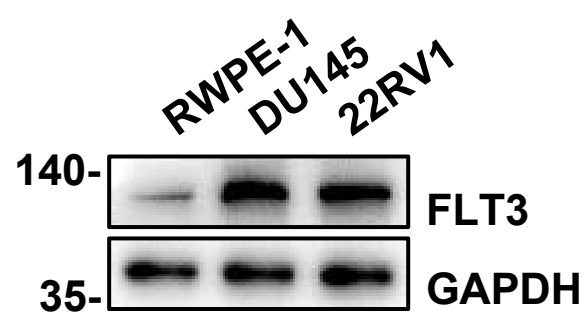

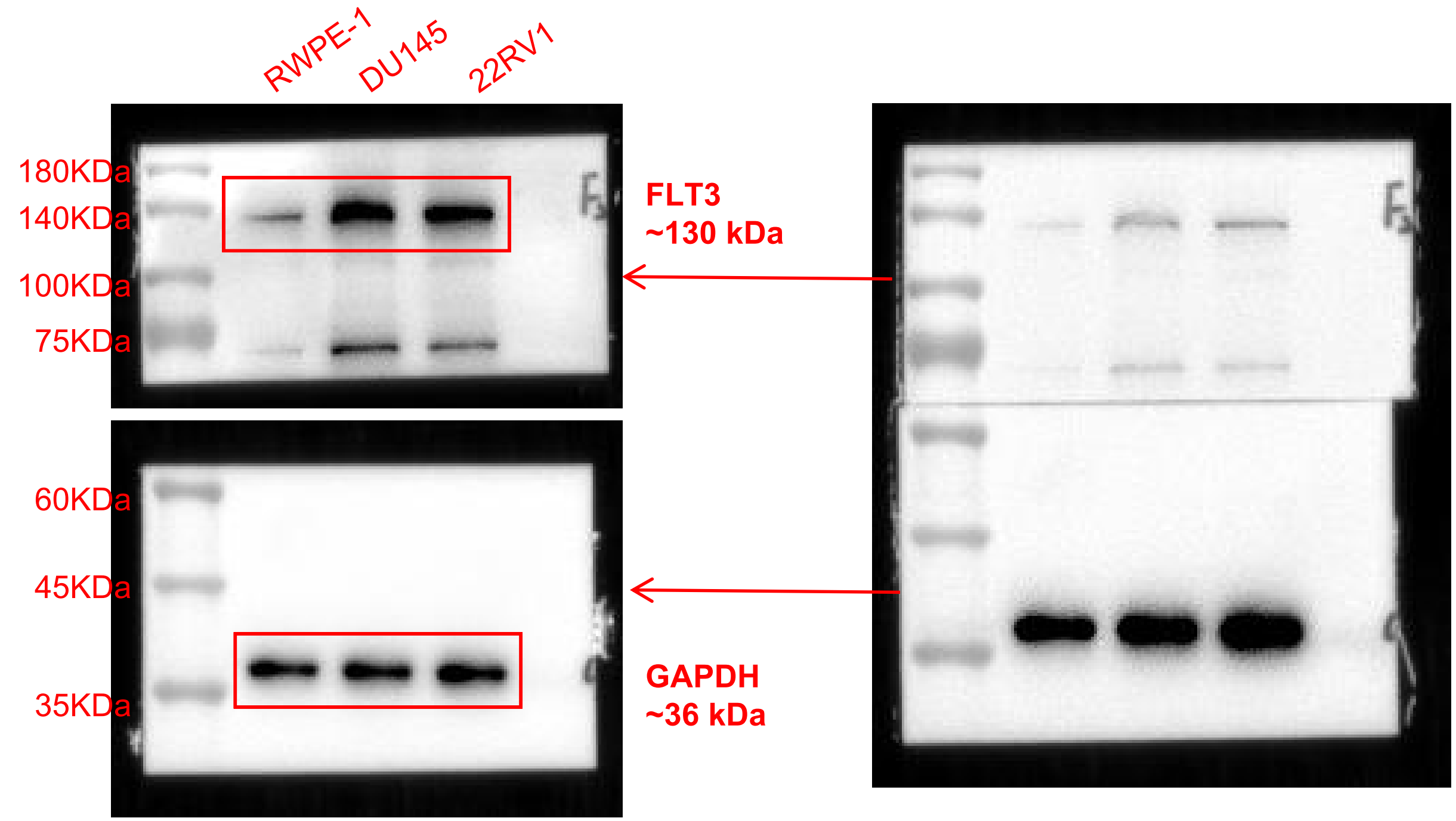

Protein ladder information used for western blot experiments

The molecular weight standard used in western blot experiments was a three-color prestained protein marker (**10–180 kDa**) purchased from GeneFist (**Shanghai GeneFist Biotechnology Co., Ltd., China; Cat. No. GF6616-10**). The marker covers a molecular weight range of 10–180 kDa and was used for molecular weight estimation of detected proteins.

Fig2E: DU145

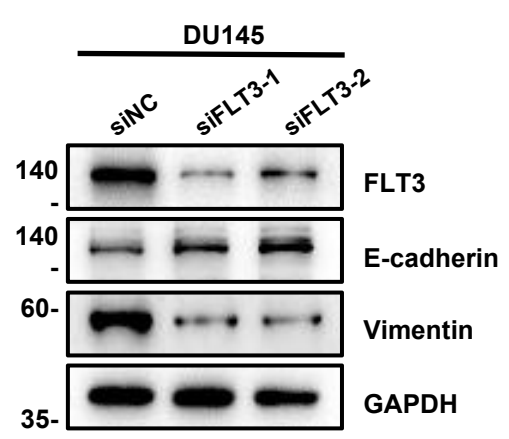
DU145
siNC
siFLT3-1
siFLT3-2
140
FLT3
140
E-cadherin
60-
Vimentin
35-
GAPDH

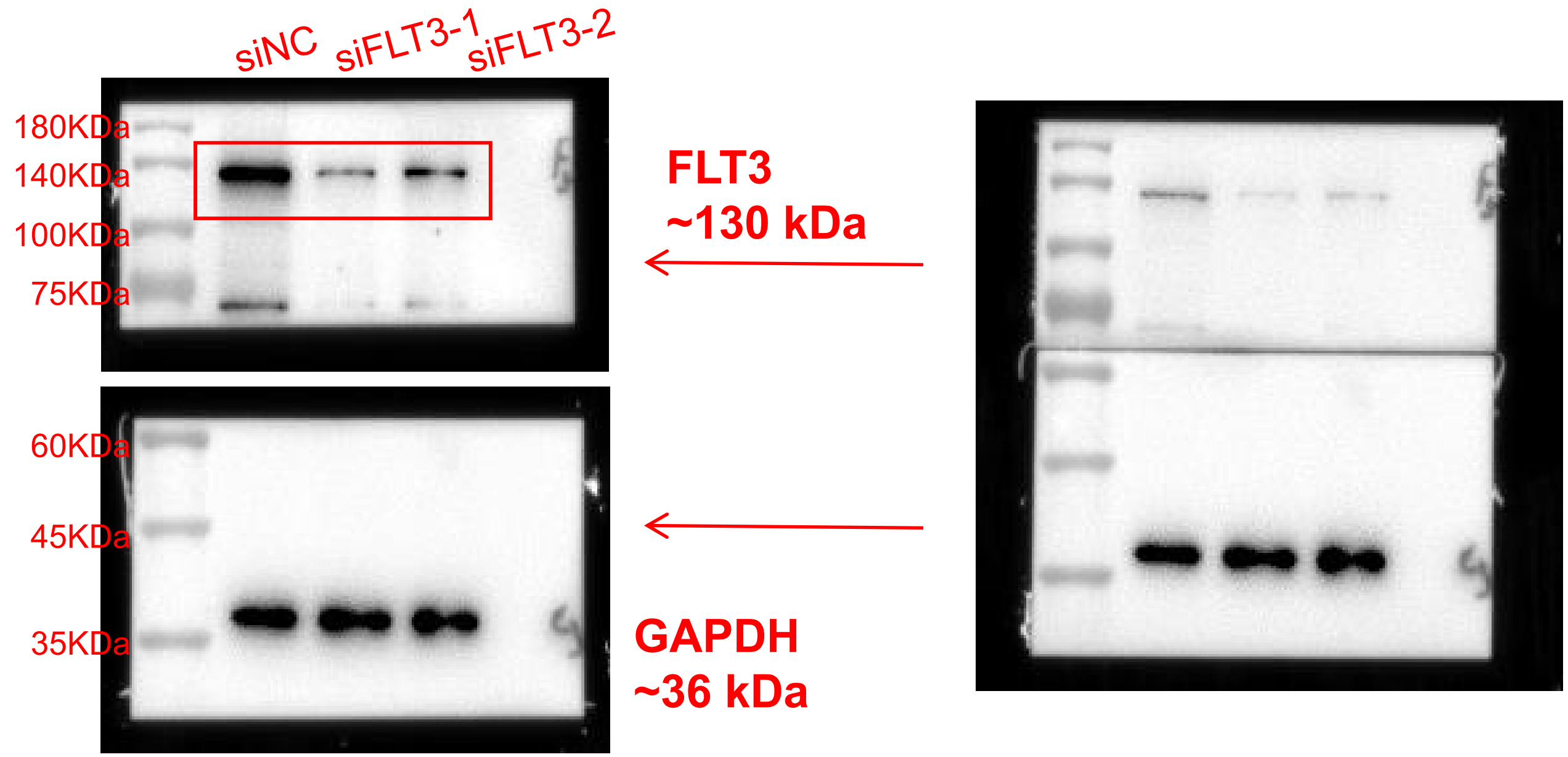
siNC
siFLT3-1
siFLT3-2
180KDa
140KDa
100KDa
75KDa
FLT3
~130 kDa
60KDa
45KDa
35KDa
GAPDH
~36 kDa

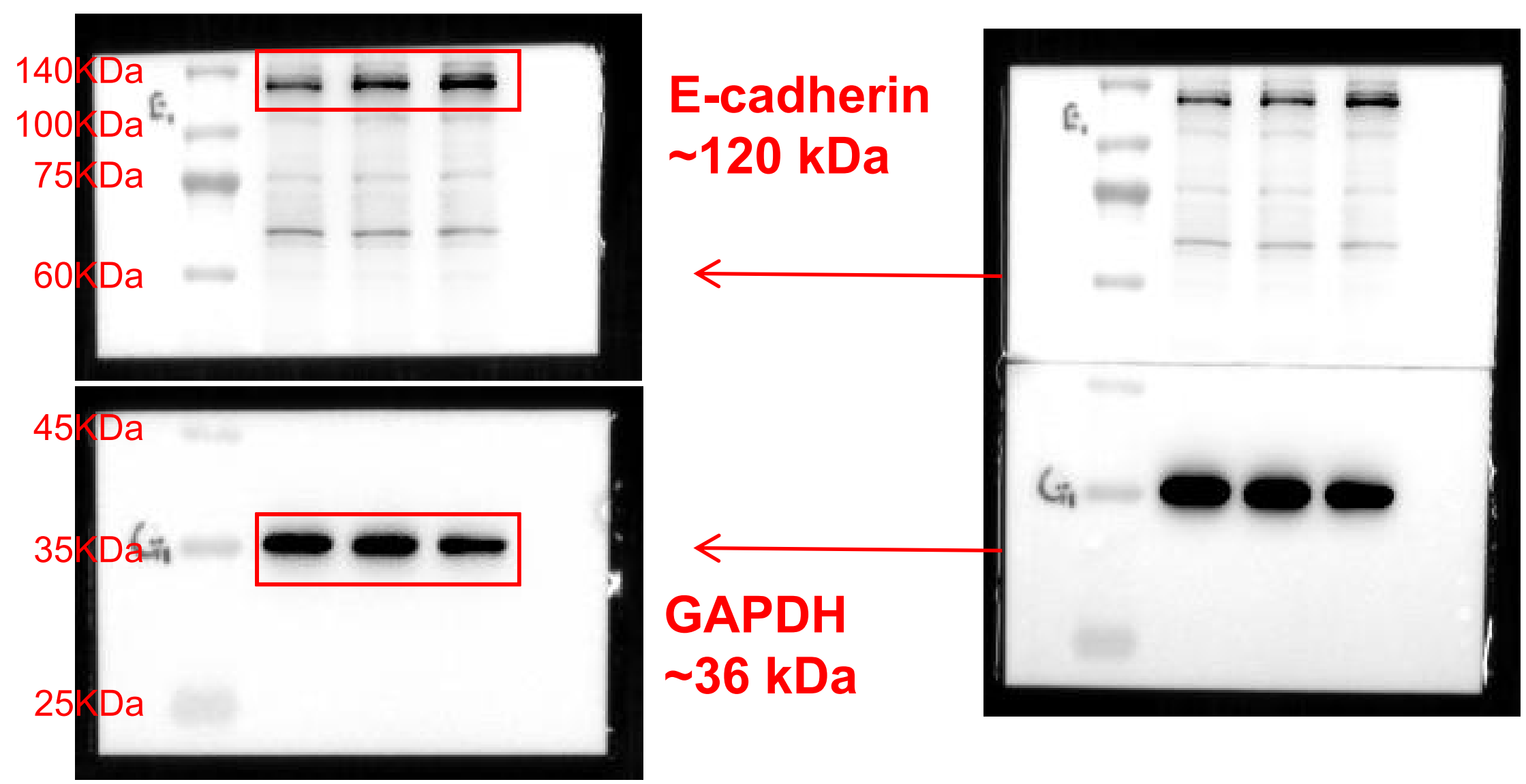
140KDa
100KDa
75KDa
60KDa
E-cadherin
~120 kDa
45KDa
35KDa
25KDa
GAPDH
~36 kDa

Fig2E: DU145

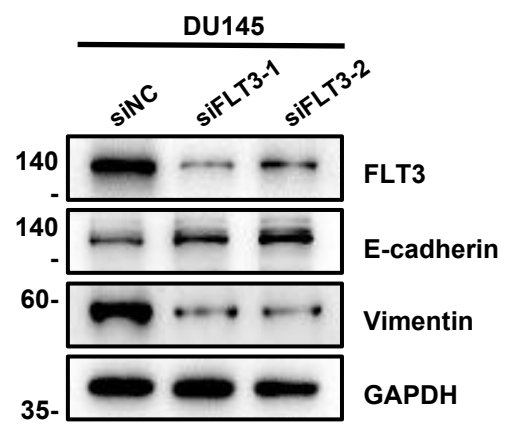
DU145
siNC
siFLT3-1
siFLT3-2
140
FLT3
140
E-cadherin
60-
Vimentin
GAPDH
35-

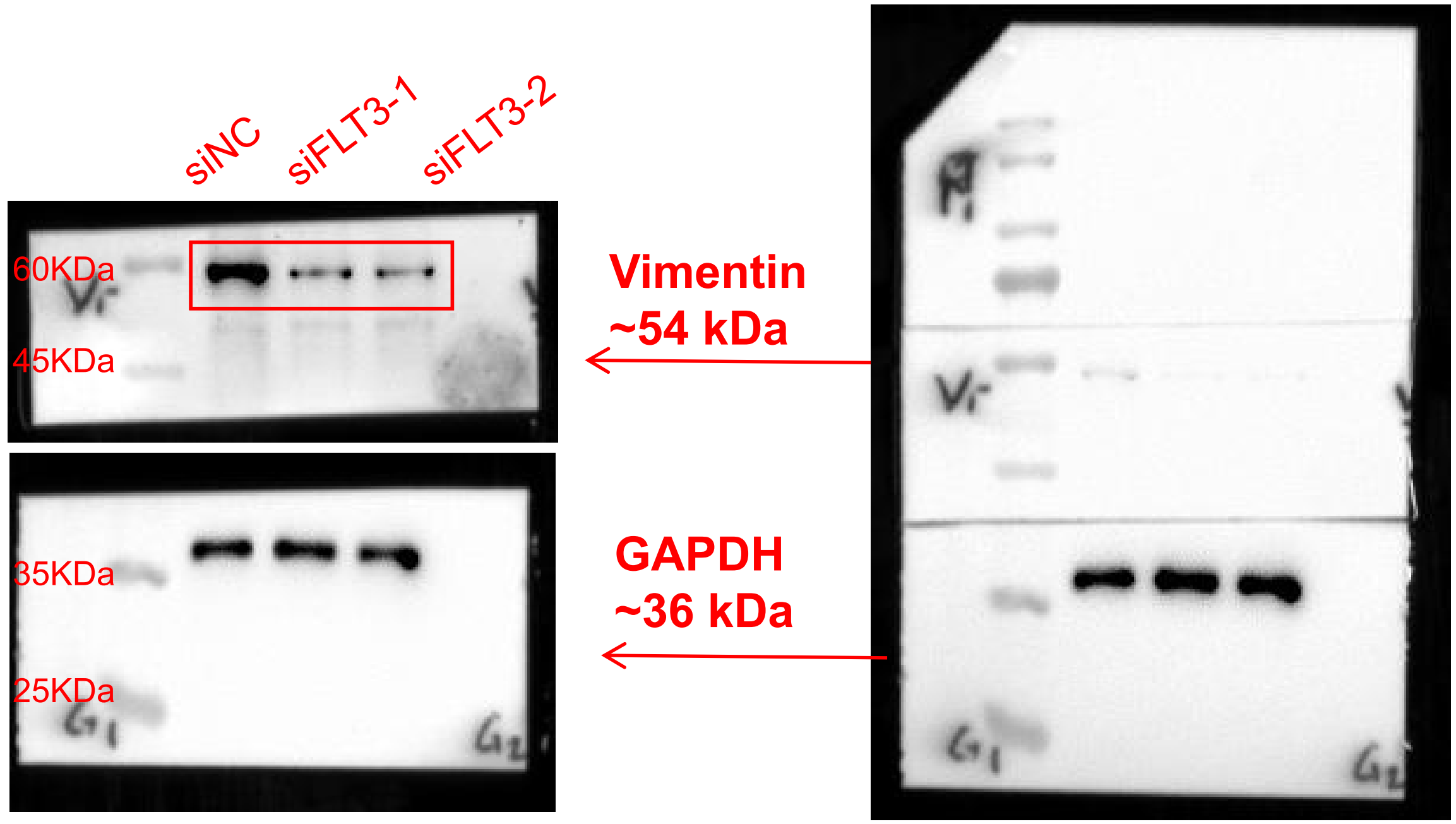
siNC
siFLT3-1
siFLT3-2
60KDa
45KDa
Vimentin
~54 kDa
35KDa
25KDa
GAPDH
~36 kDa

Fig2E: 22RV1

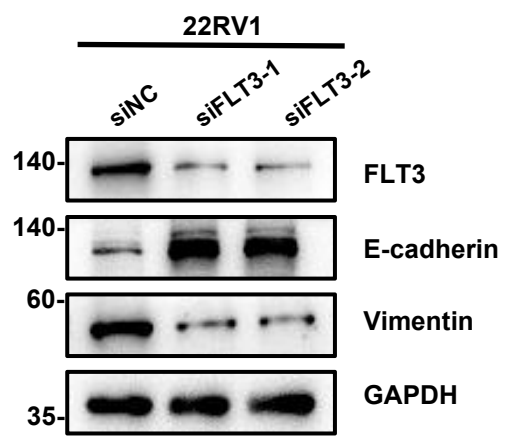
22RV1
siNC
siFLT3-1
siFLT3-2
140-
FLT3
140-
E-cadherin
60-
Vimentin
35-
GAPDH

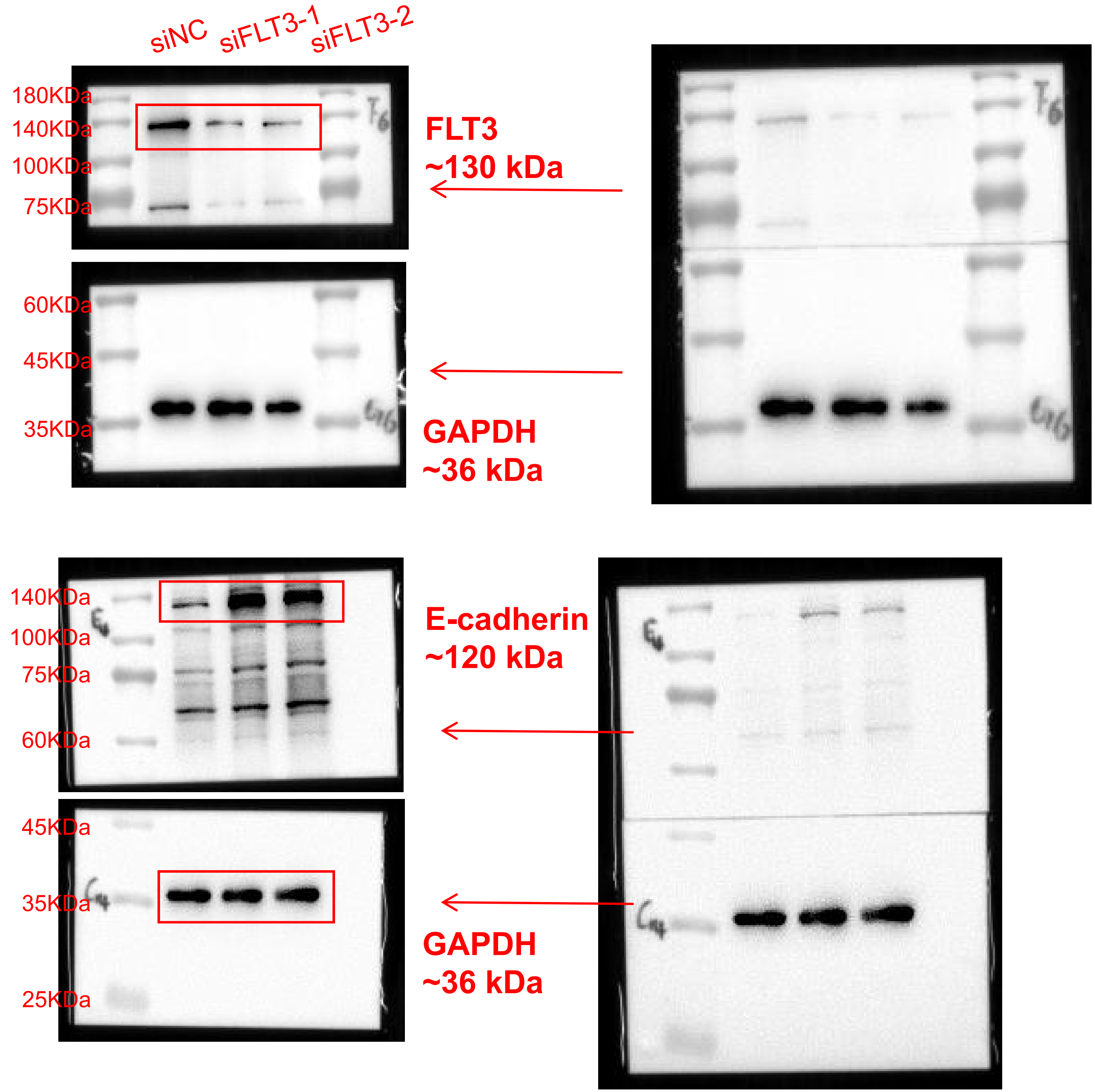
siNC
siFLT3-1
siFLT3-2
180KDa
140KDa
100KDa
75KDa
FLT3
~130 kDa
60KDa
45KDa
35KDa
GAPDH
~36 kDa
140KDa
100KDa
75KDa
60KDa
E-cadherin
~120 kDa
45KDa
35KDa
25KDa
GAPDH
~36 kDa

Fig2E: 22RV1

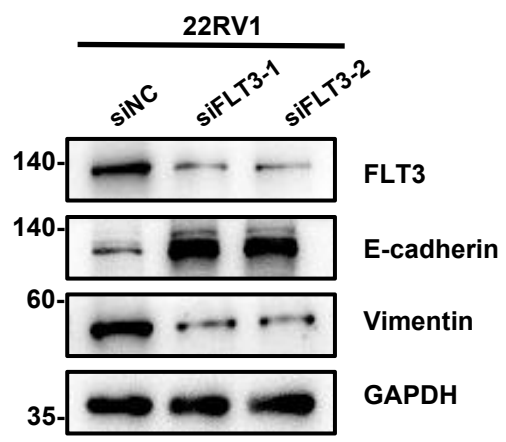
22RV1
siNC
siFLT3-1
siFLT3-2
140-
FLT3
140-
E-cadherin
60-
Vimentin
35-
GAPDH

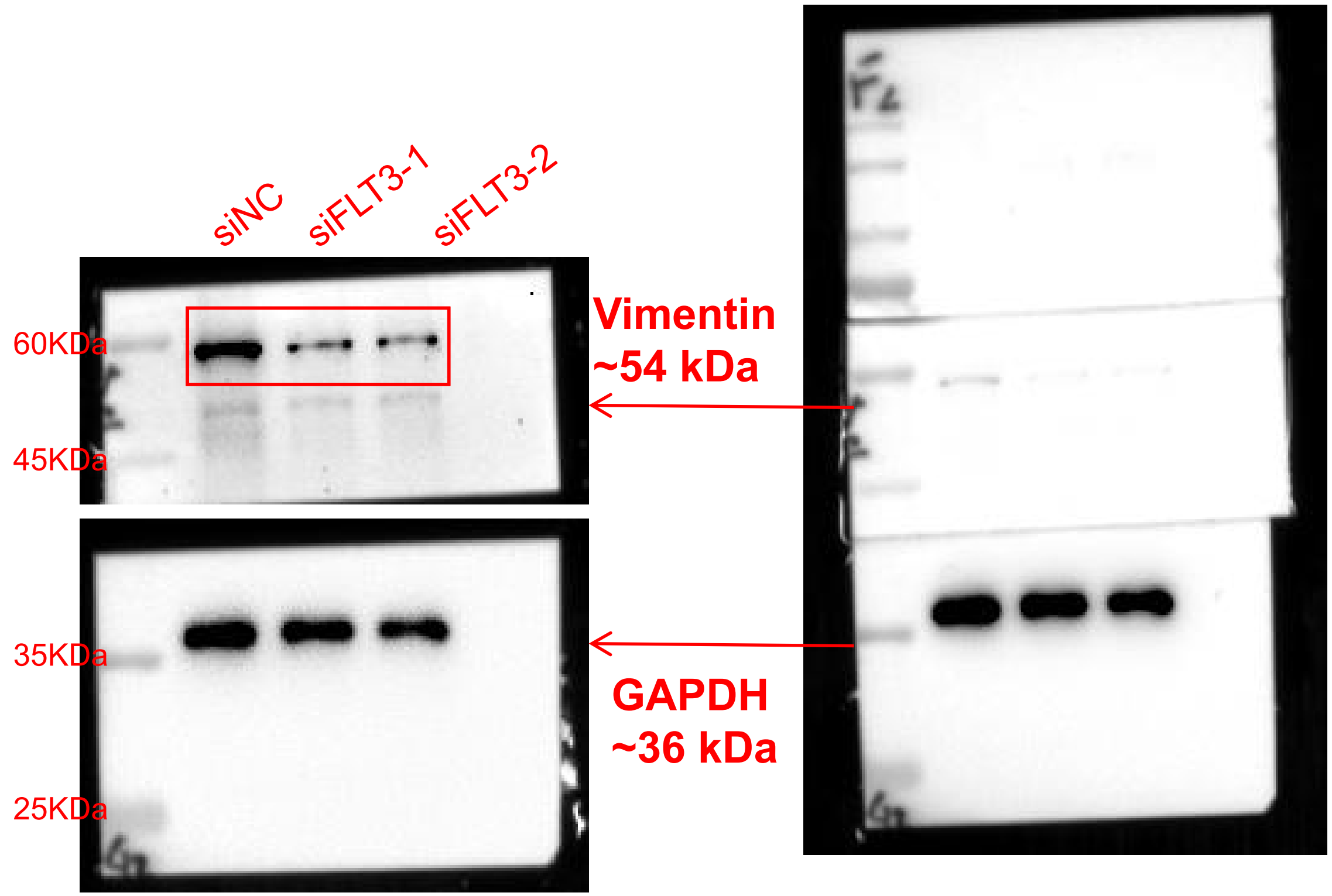
siNC
siFLT3-1
siFLT3-2
60KDa
45KDa
35KDa
25KDa
Vimentin
~54 kDa
GAPDH
~36 kDa
