# Supplementary material for: FLT3 expression and immune-associated transcriptional signatures in prostate cancer
Source: Front Oncol. 2026 Oct 23;16:1970520. doi: 10.3389/fonc.2026.1970520 (PMC13739570; doi:10.3389/fonc.2026.1970520)

Uncropped and unprocessed western blot images corresponding to Figure 2.

Fig2A FLT3,GAPDH

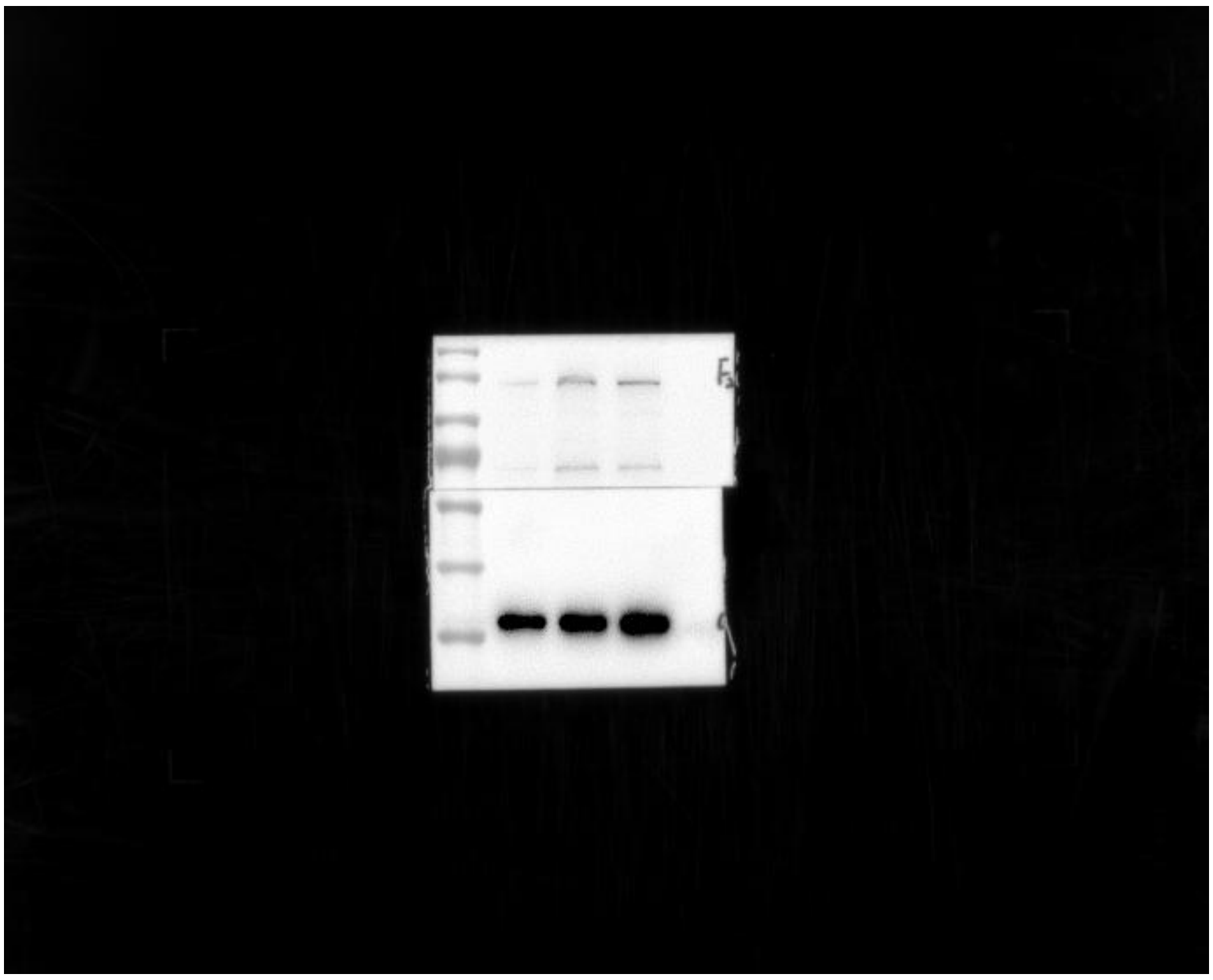

Fig2A FLT3

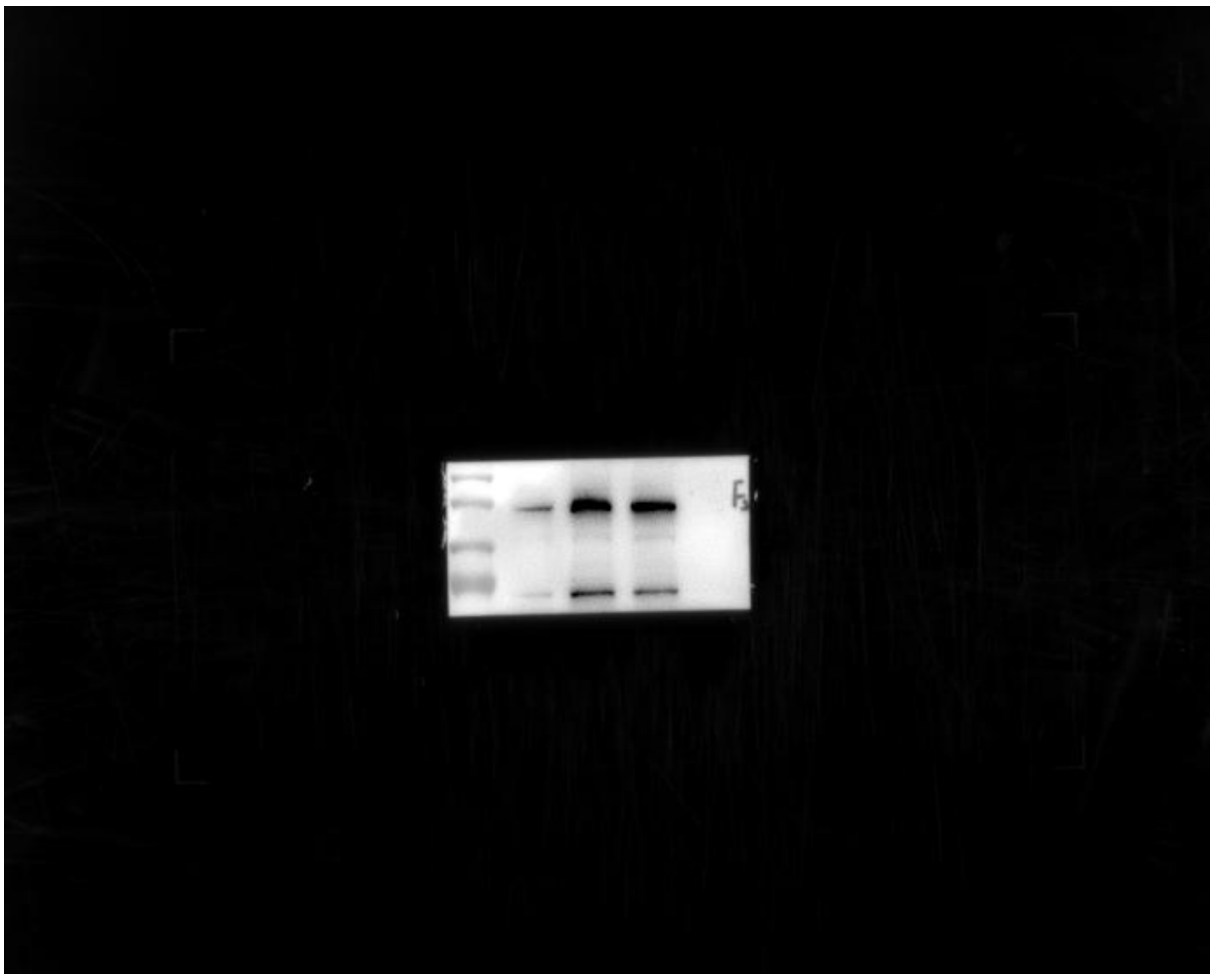

Fig2A GAPDH

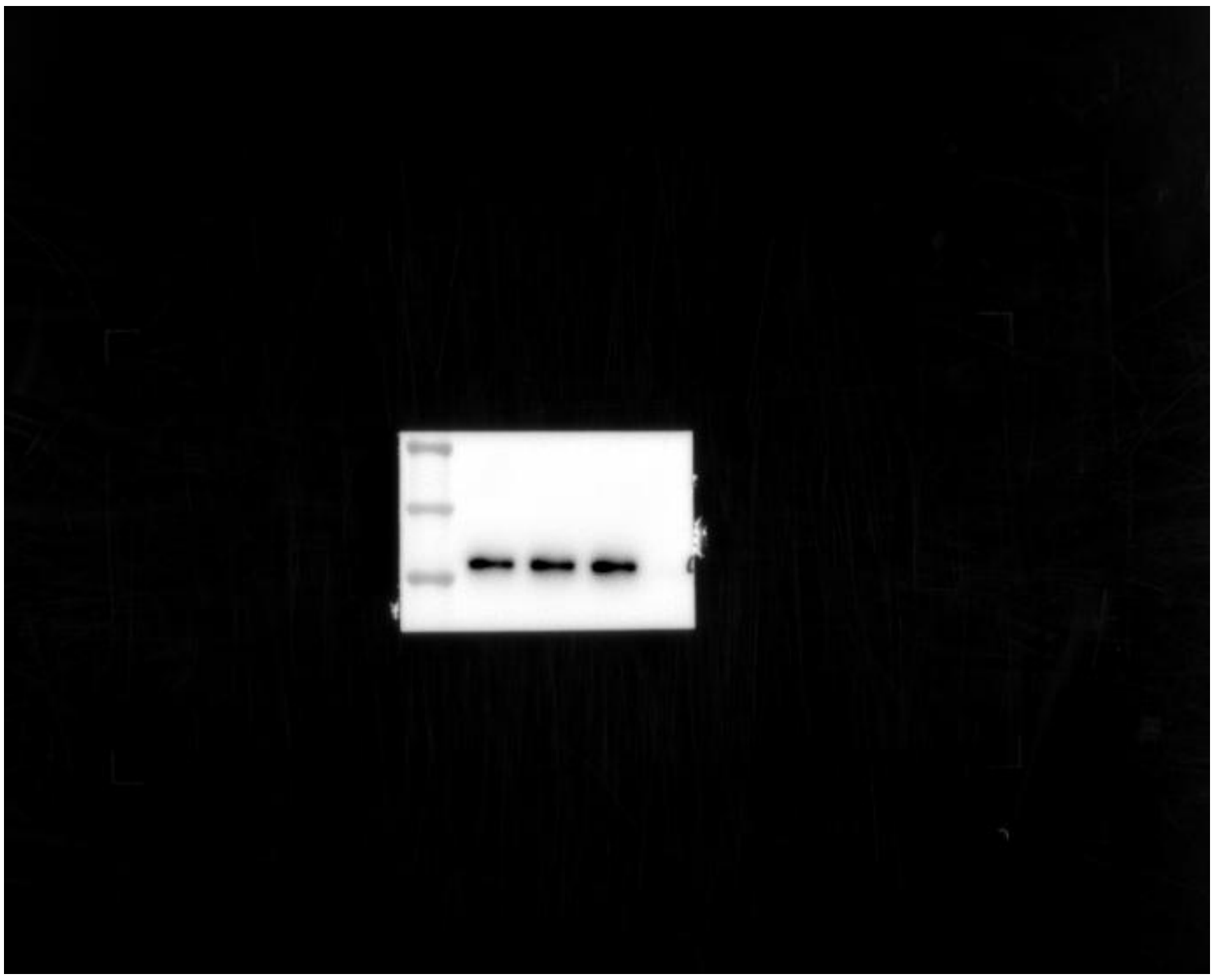

Fig2E 22RV1 Ecadherin,GAPDH

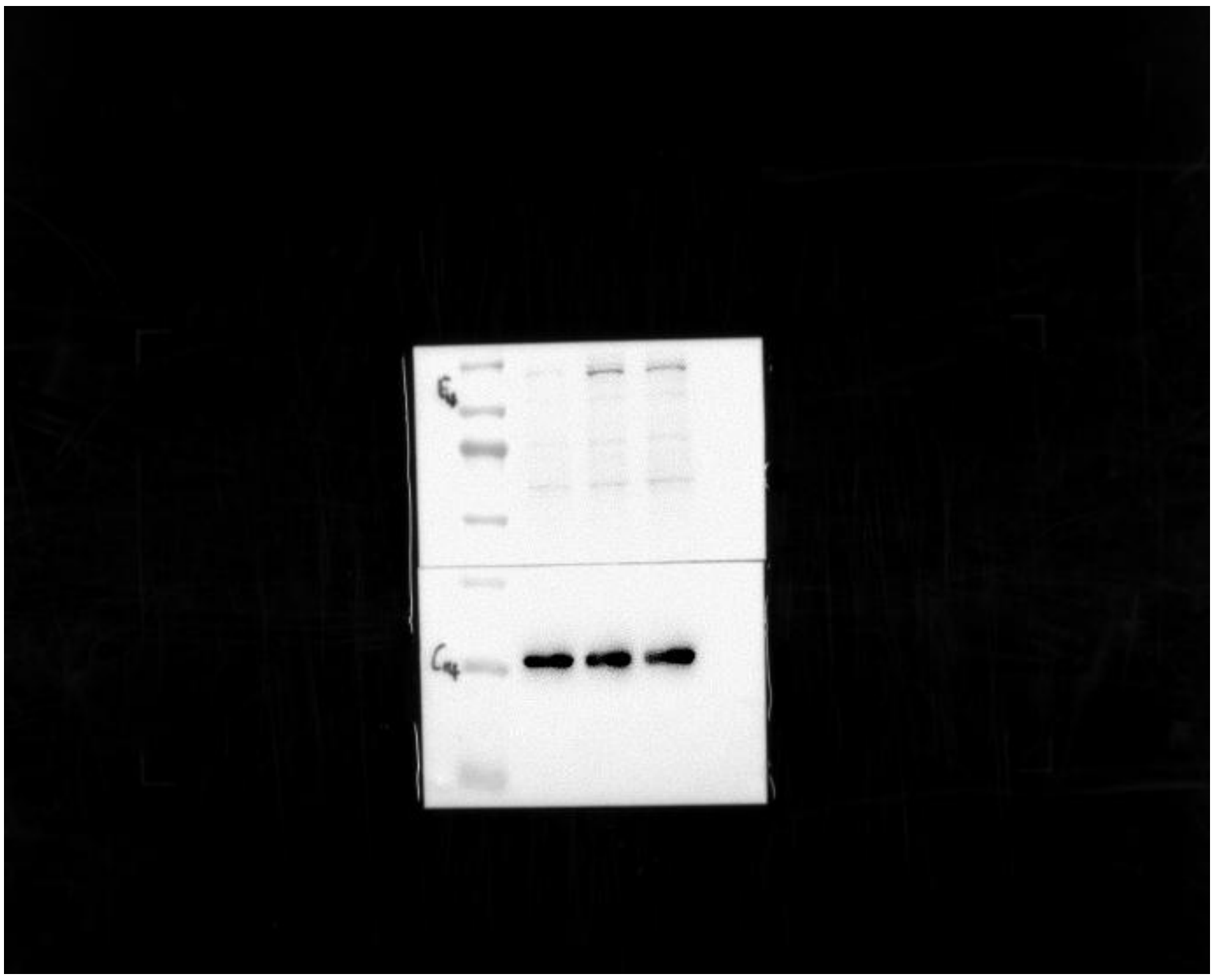

Fig2E 22RV1 Ecadherin

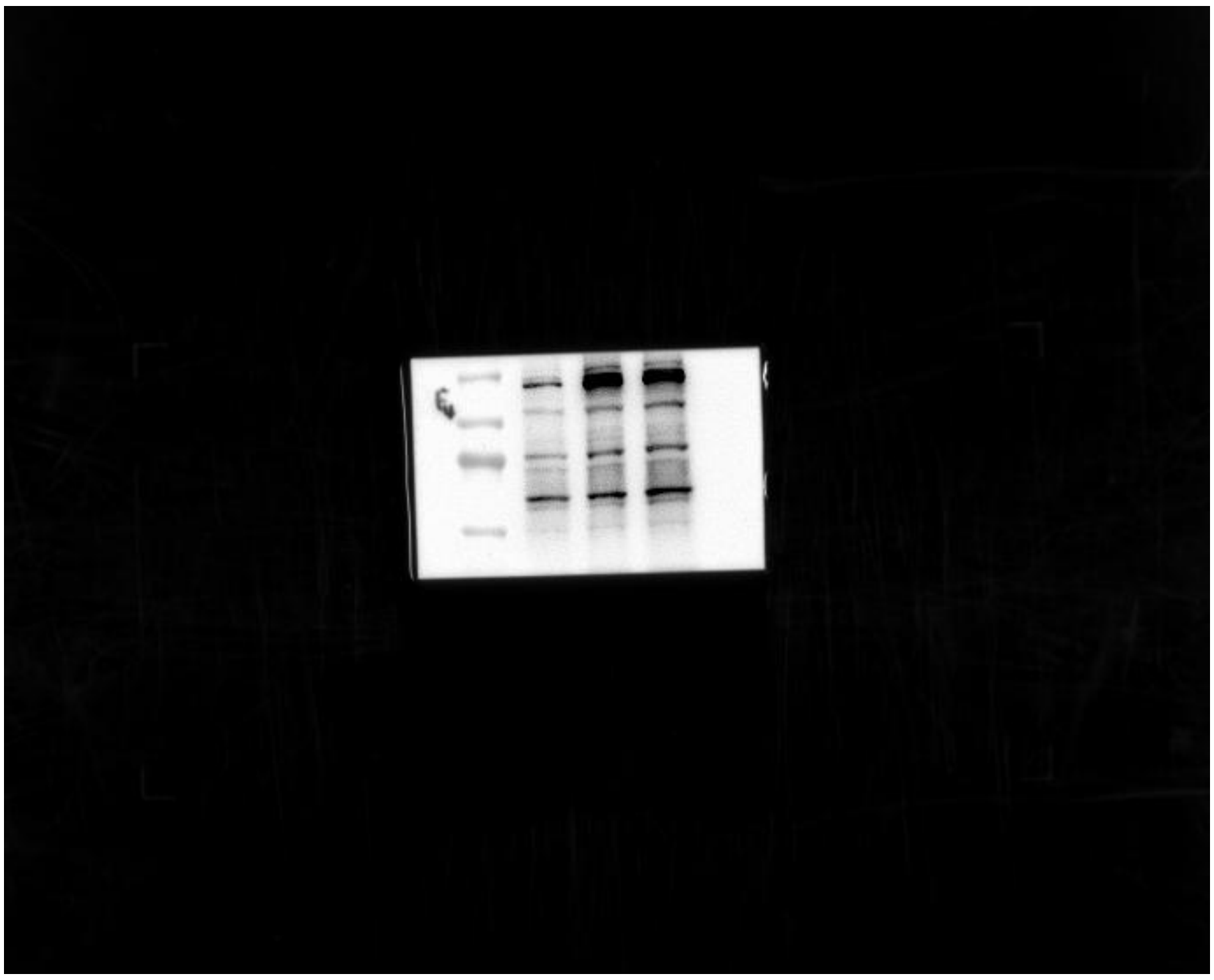

Fig2E 22RV1 FLT3,GAPDH

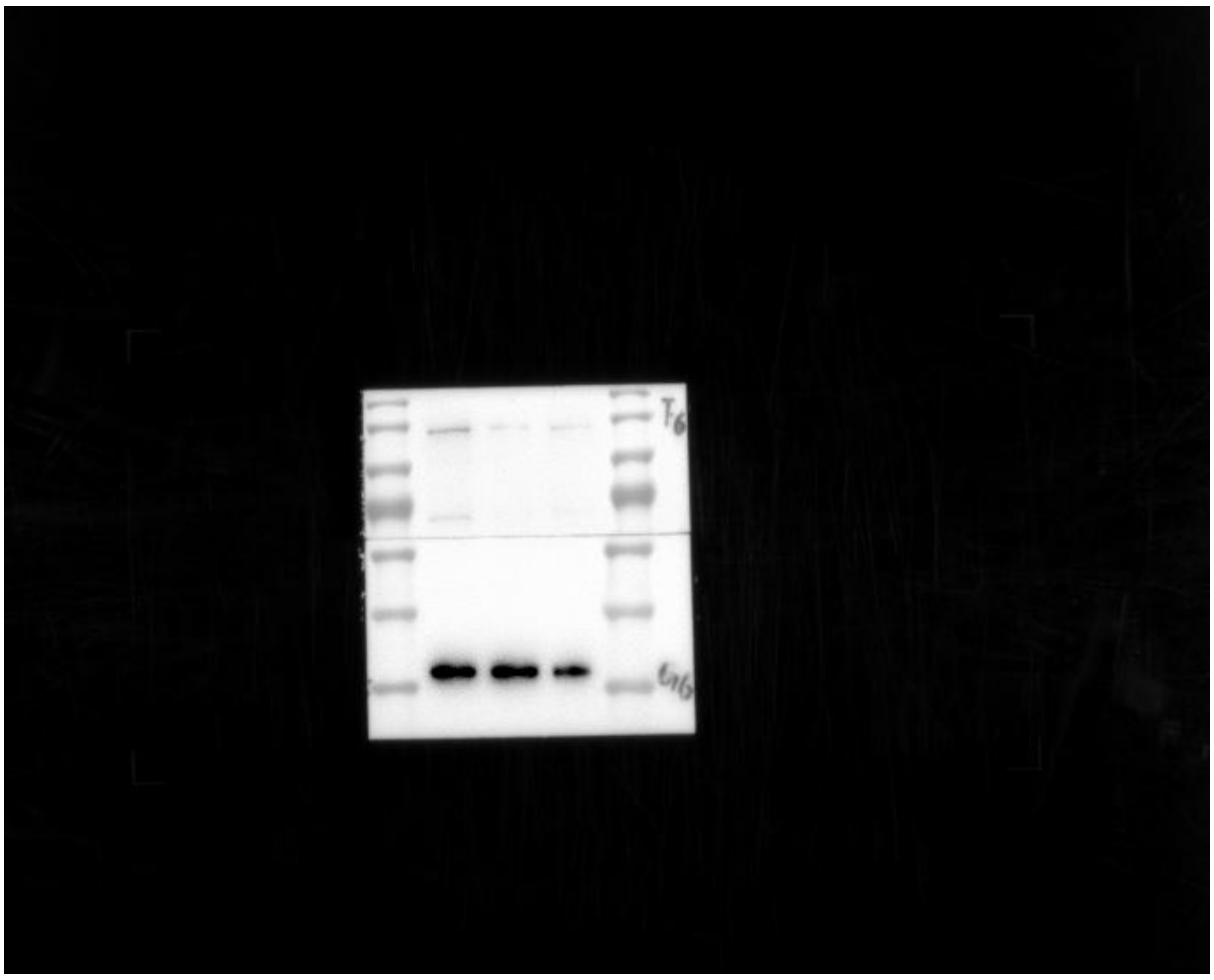

Fig2E 22RV1 FLT3,GAPDH1

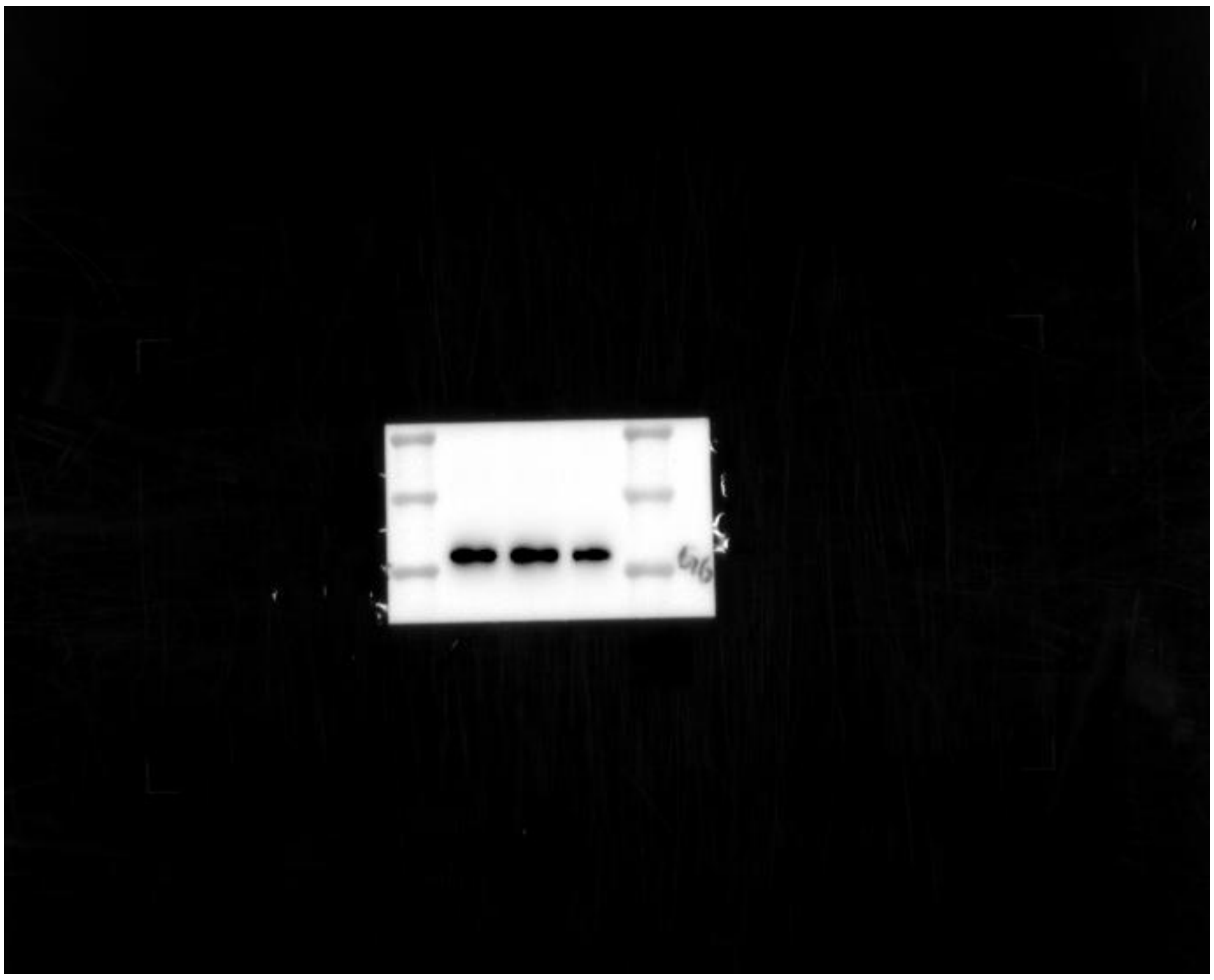

Fig2E 22RV1 FLT3

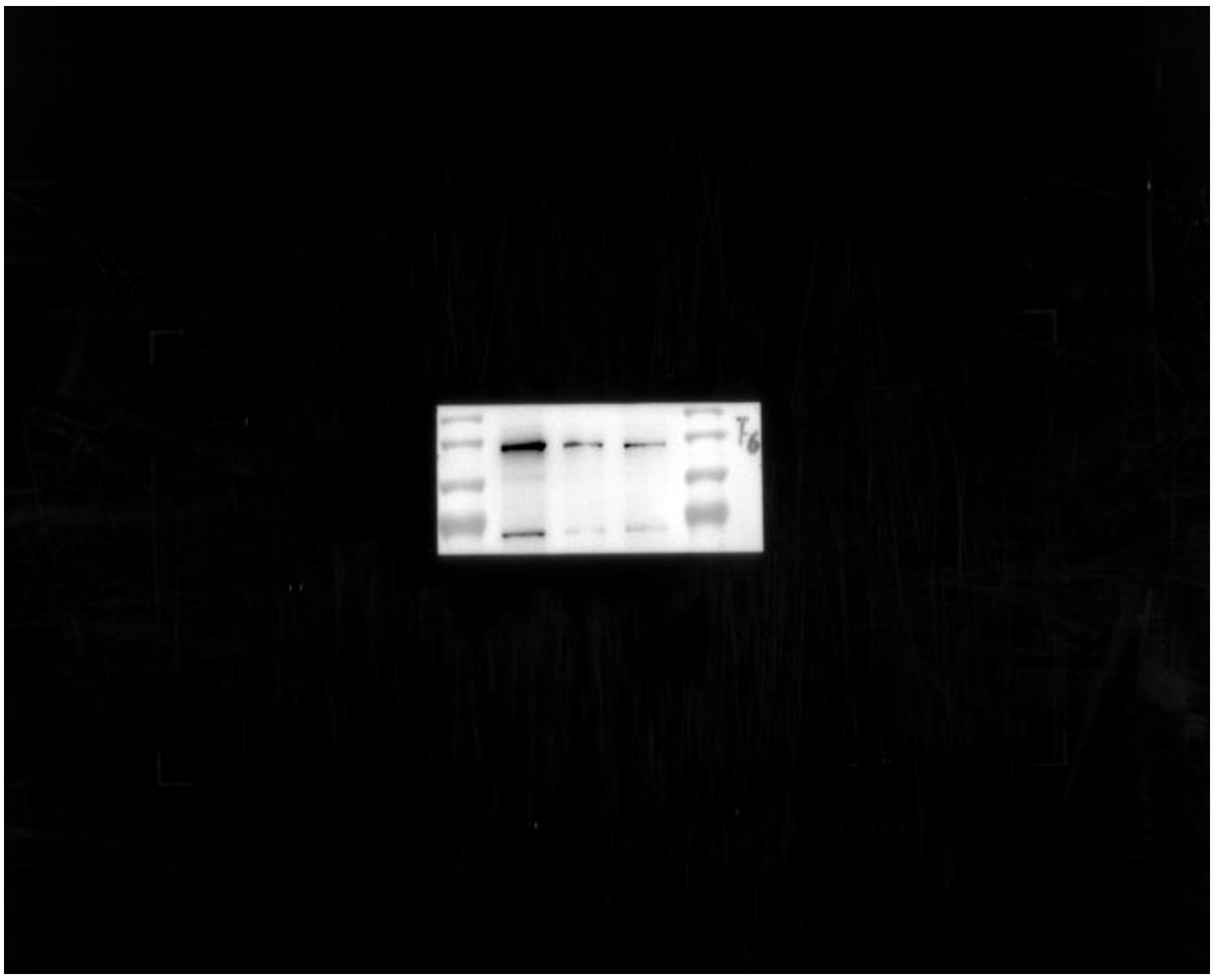

Fig2E 22RV1 GAPDH

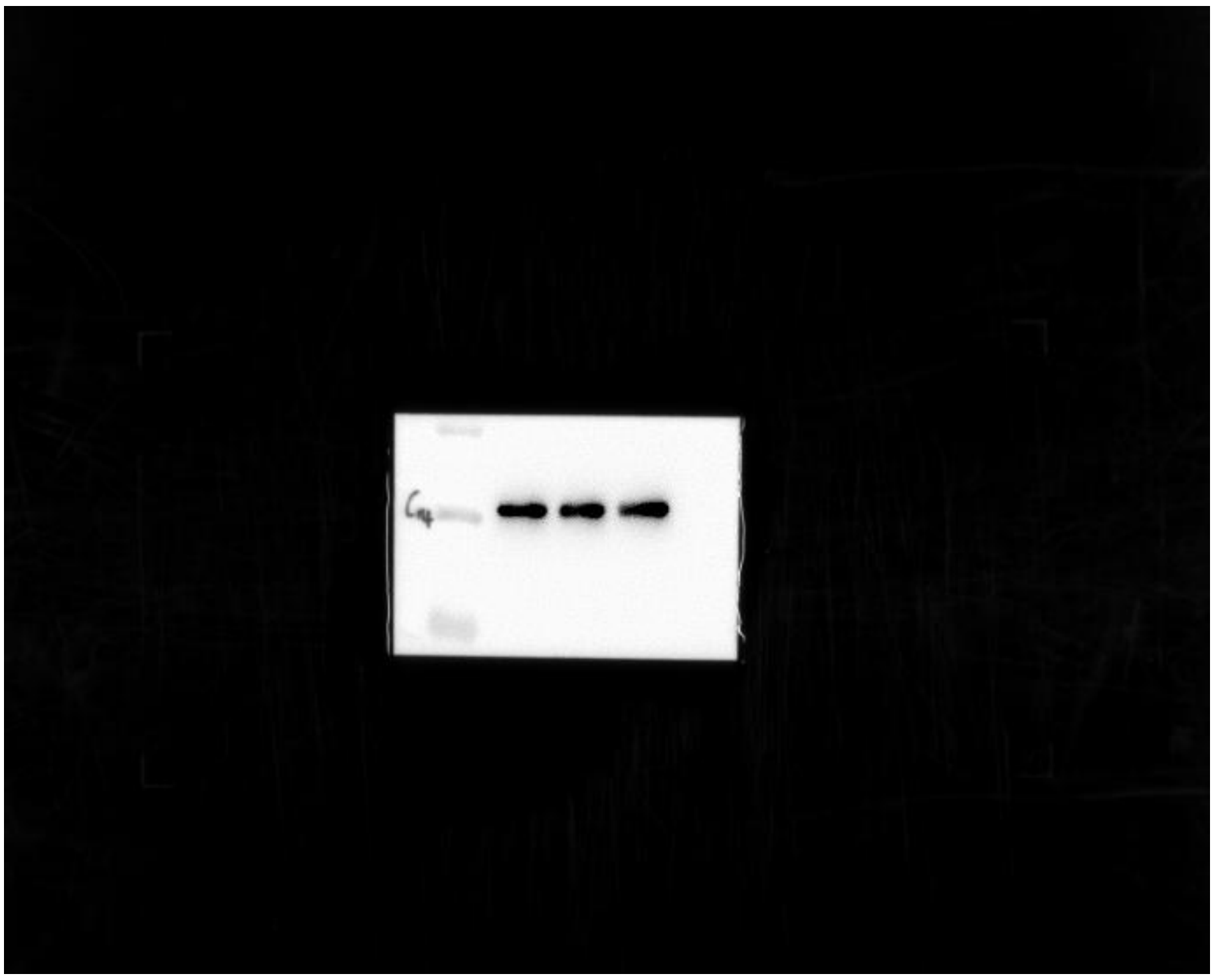

Fig2E DU145 Ecadherin,GAPDH

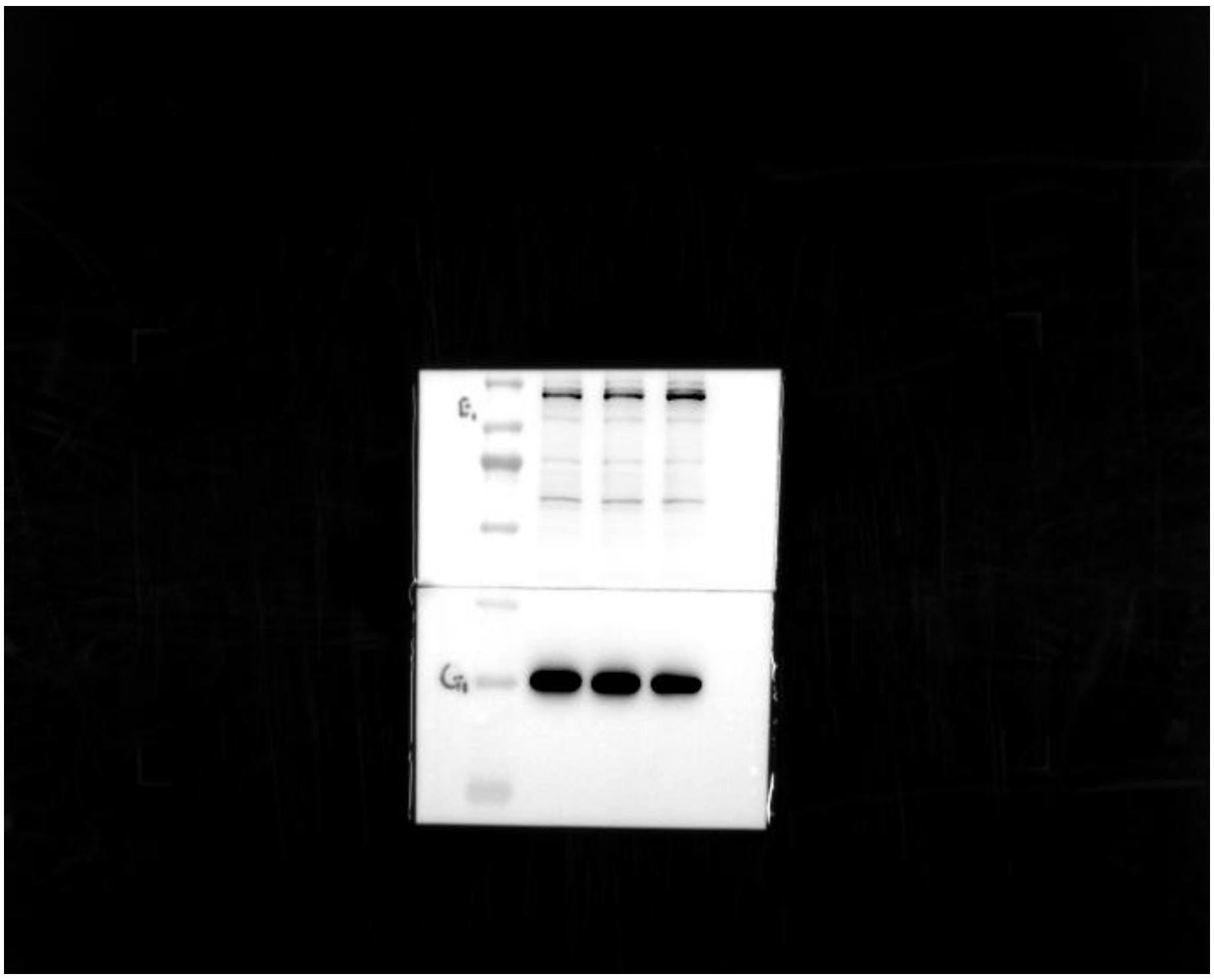

Fig2E DU145 Ecadherin

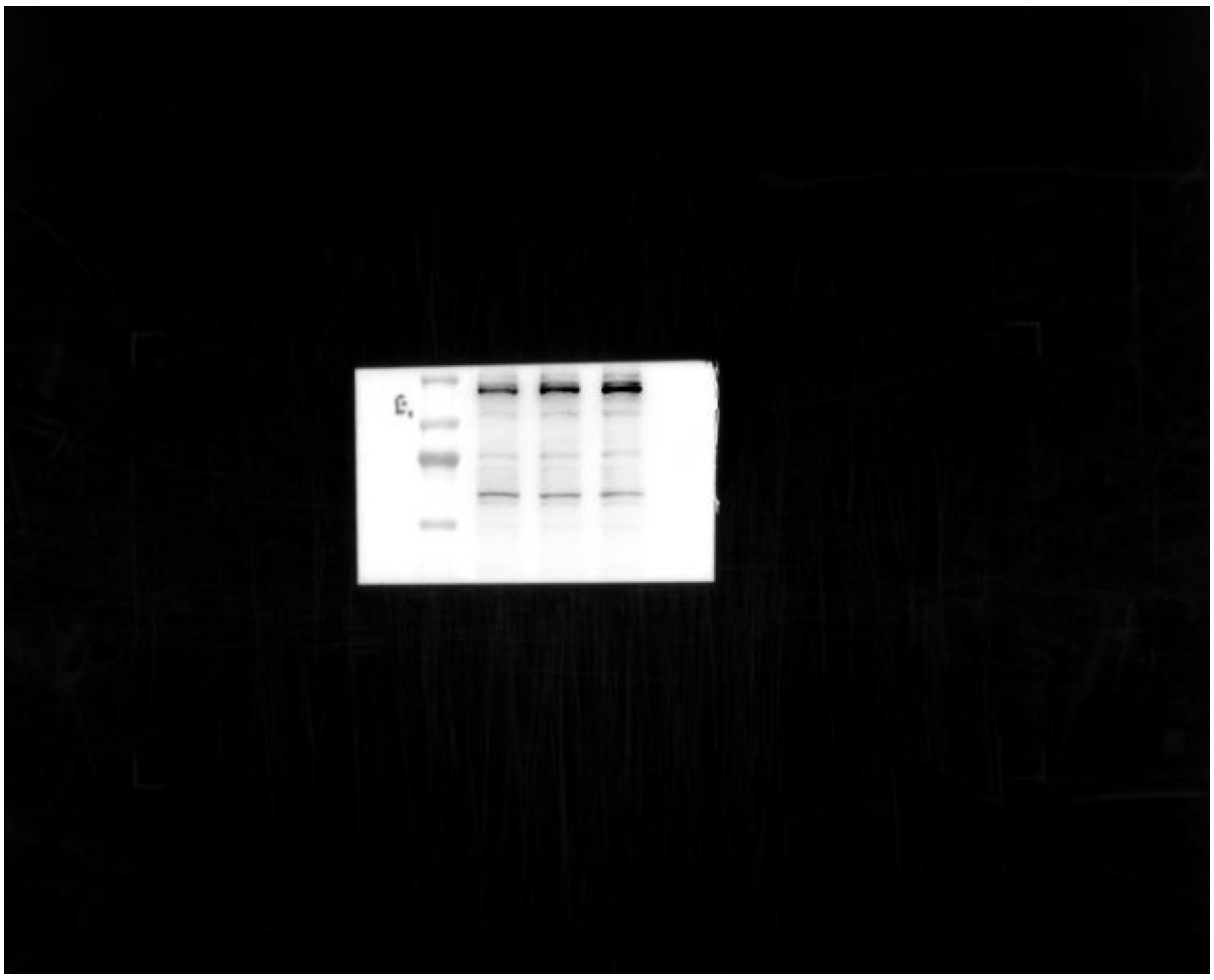

Fig2E DU145 FLT3,GAPDH

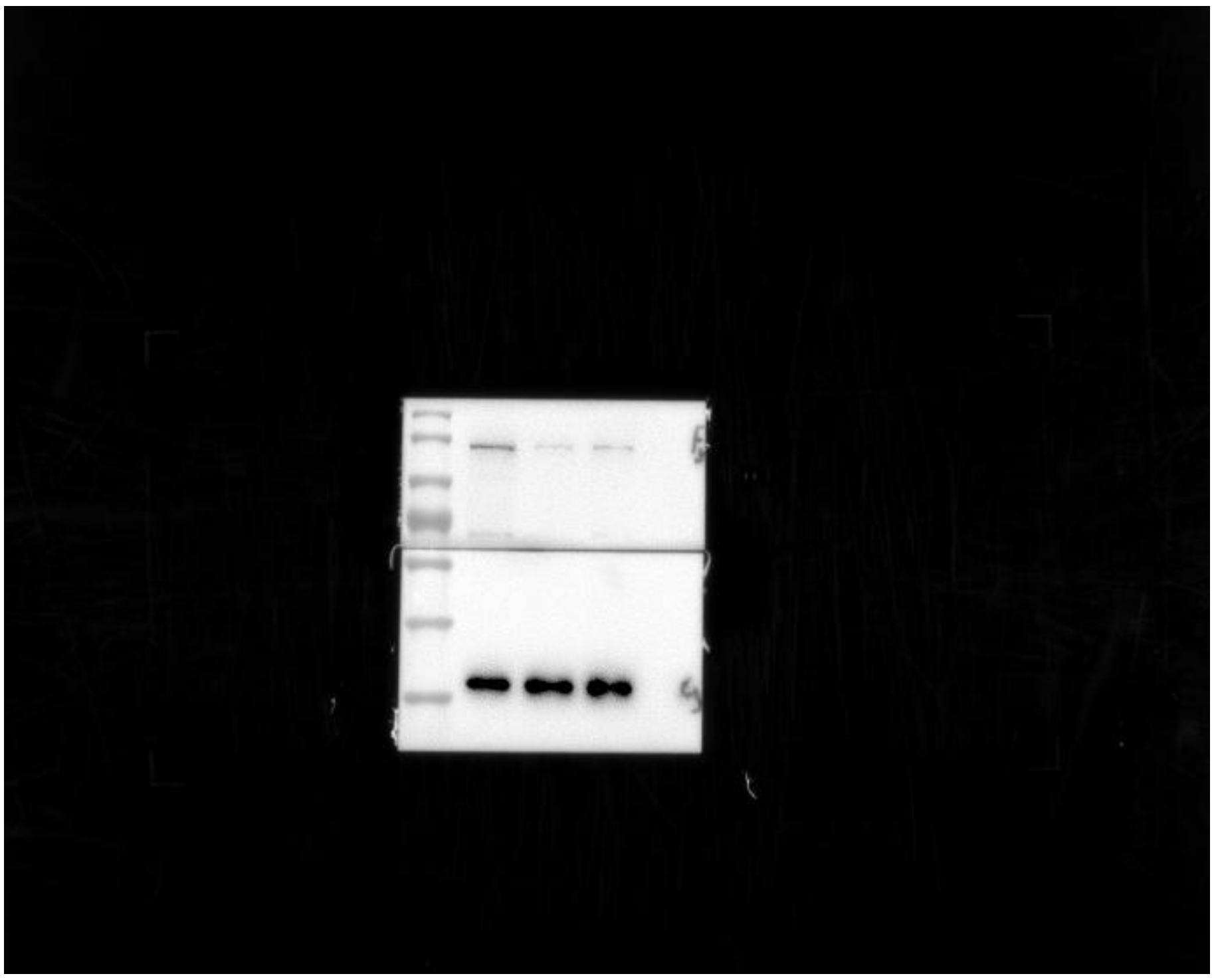

Fig2E DU145 FLT3,GAPDH1

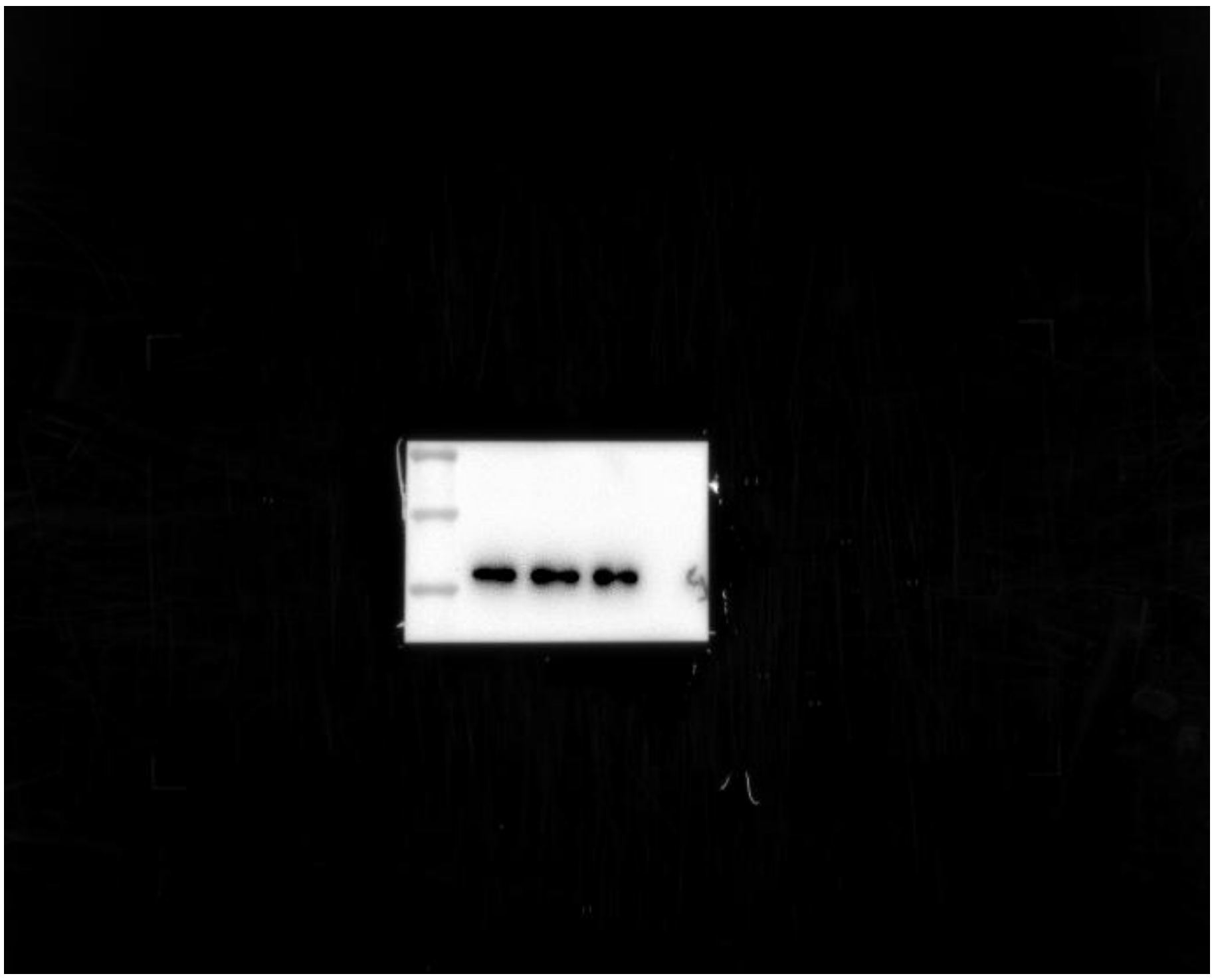

Fig2E DU145 FLT3

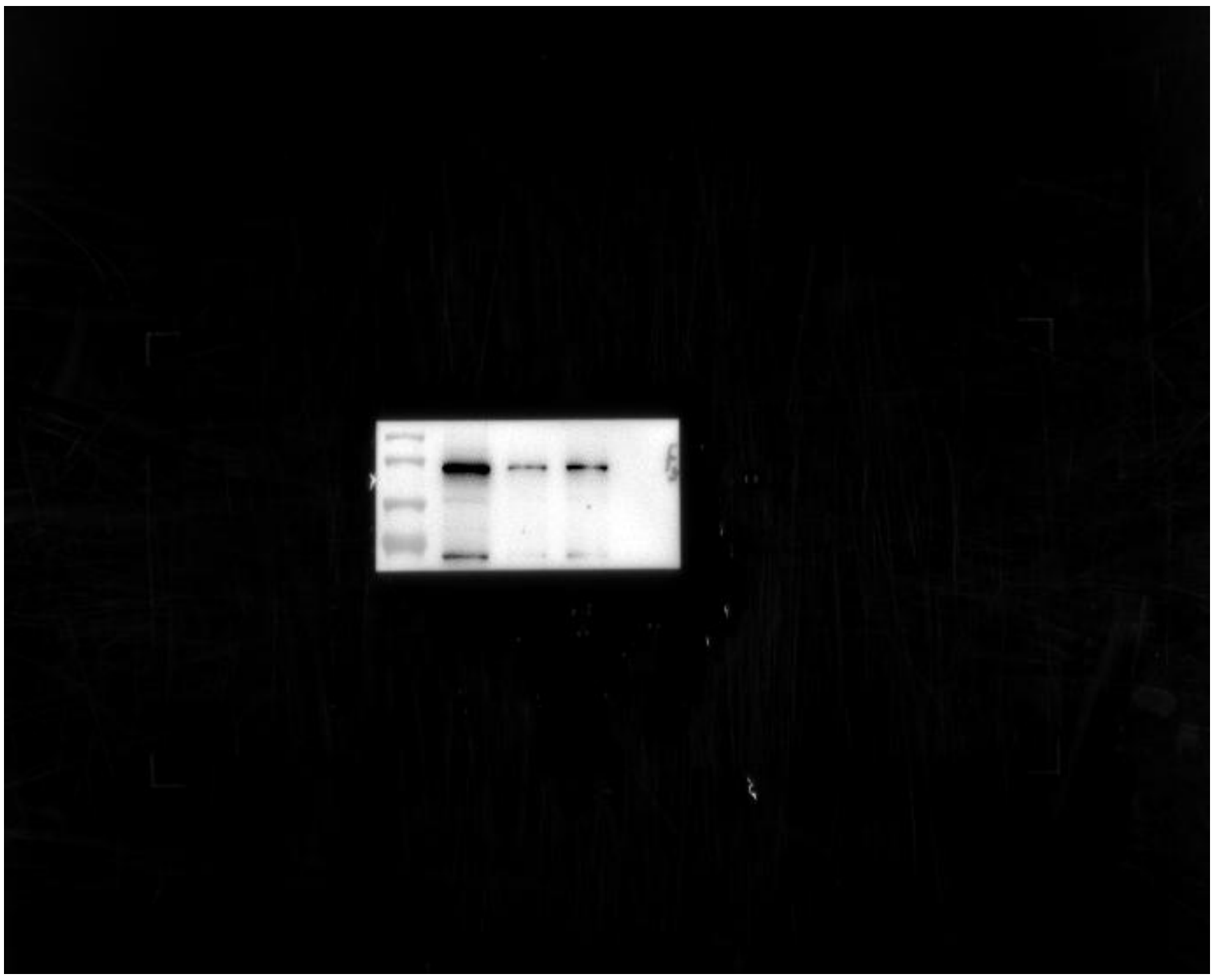

Fig2E DU145 GAPDH

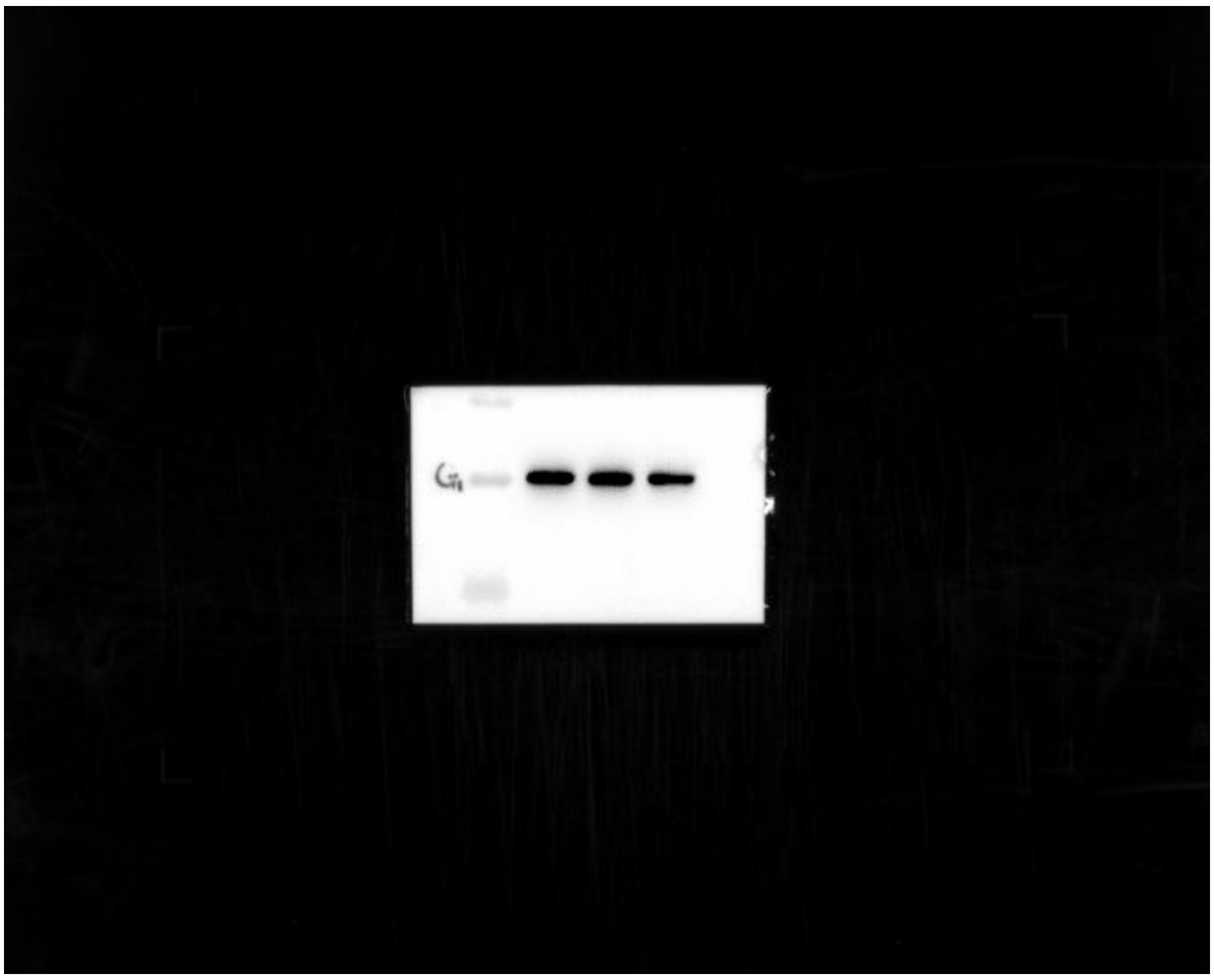

Fig2E DU145 Vimentin,GAPDH

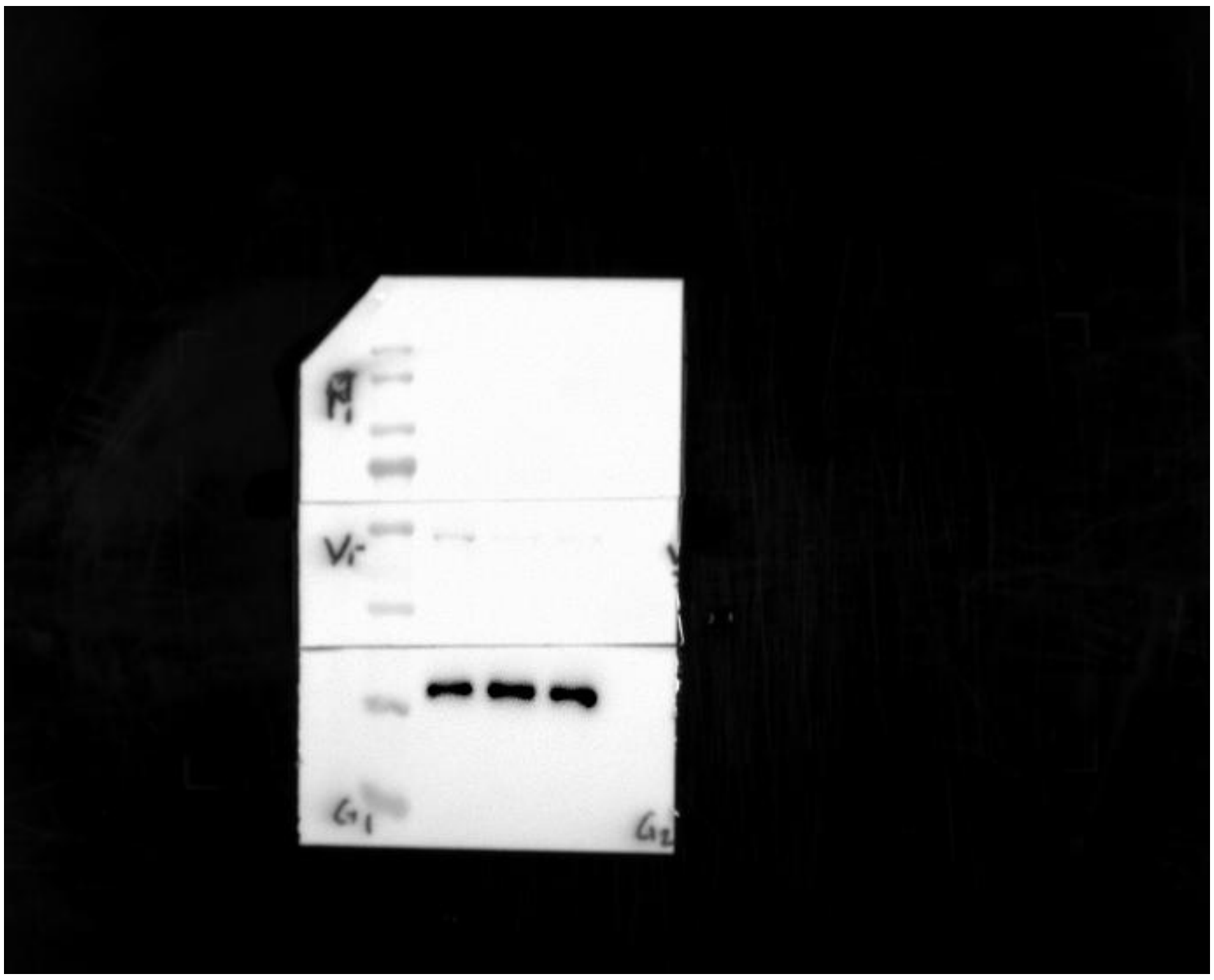

Fig2E DU145 Vimentin,GAPDH1

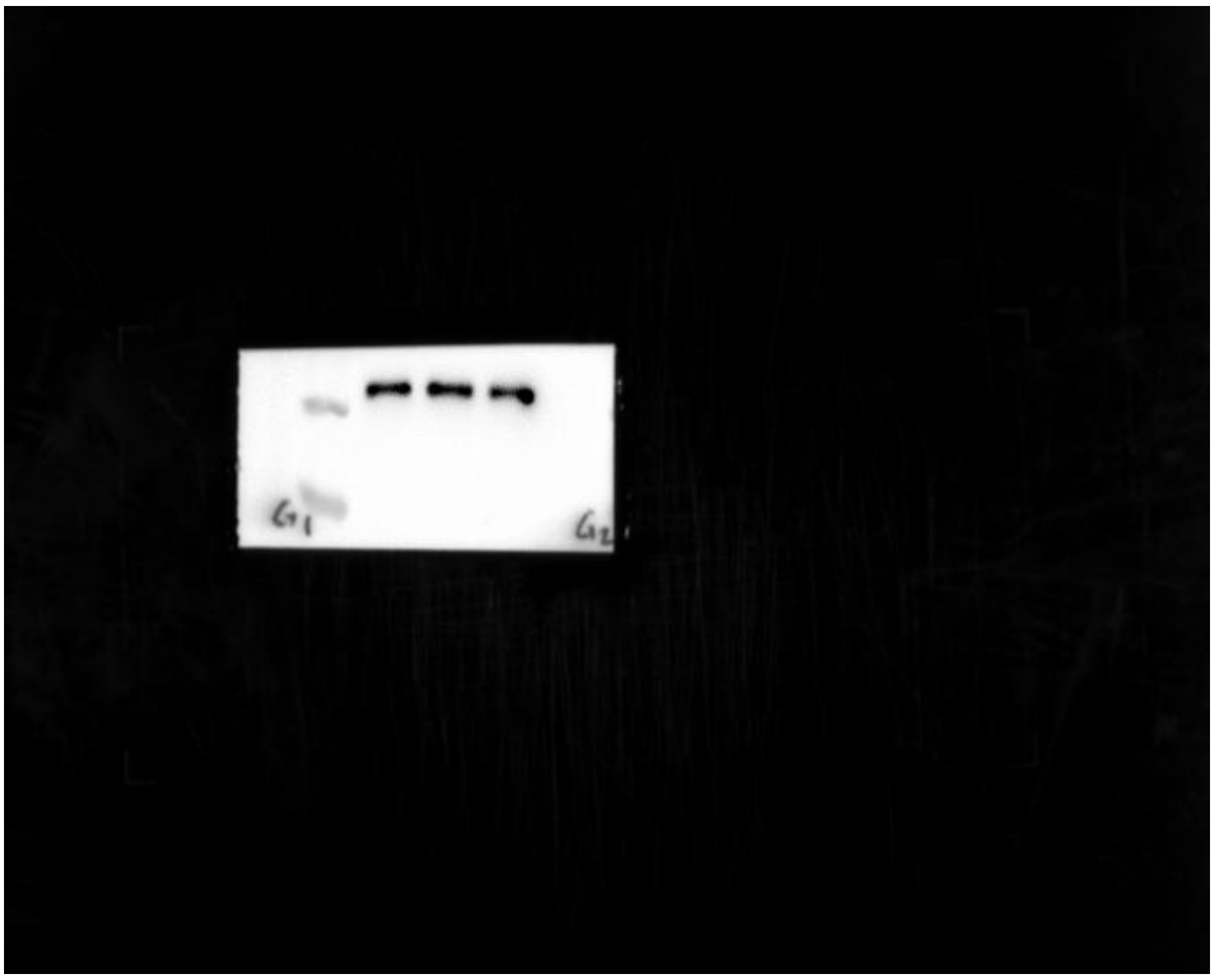

Fig2E DU145 Vimentin

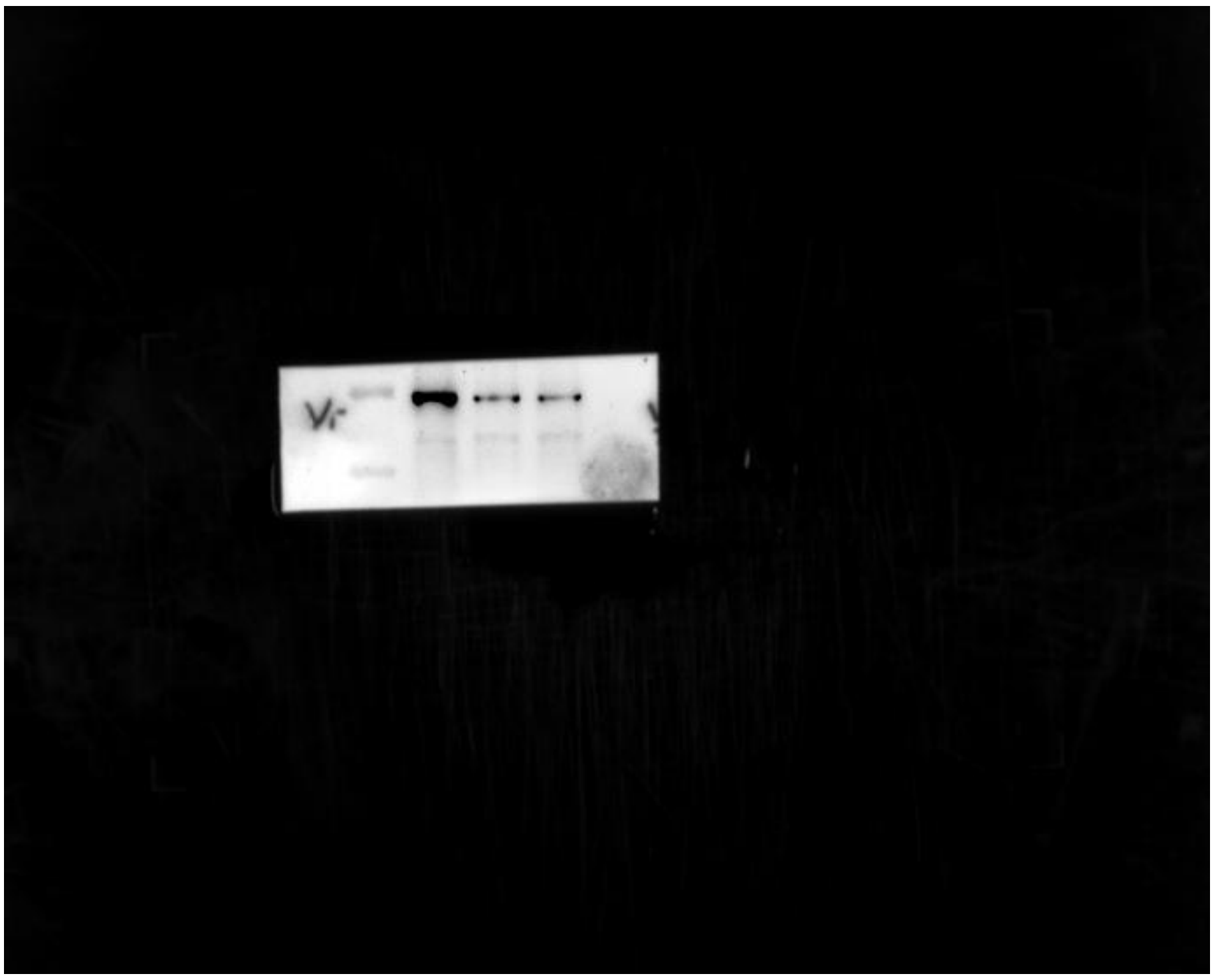

Supplement: Supplementary file 3 [file Supplementaryfile3.pdf]
